# Translating genetic and functional data into clinical practice: a series of 223 families with myotonia

**DOI:** 10.1093/brain/awab344

**Published:** 2021-09-16

**Authors:** Karen Suetterlin, Emma Matthews, Richa Sud, Samuel McCall, Doreen Fialho, James Burge, Dipa Jayaseelan, Andrea Haworth, Mary G Sweeney, Dimitri M Kullmann, Stephanie Schorge, Michael G Hanna, Roope Männikkö

**Affiliations:** 1 MRC International Centre for Genomic Medicine in Neuromuscular Diseases, Department of Neuromuscular Disease, UCL Queen Square Institute of Neurology, London, UK; 2 AGE Research Group, NIHR Newcastle Biomedical Research Centre, Newcastle-upon-Tyne Hospitals NHS Foundation Trust and Newcastle University, Newcastle-upon-Tyne, UK; 3 Atkinson Morley Neuromuscular Centre, Department of Neurology, St Georges University Hospitals NHS Foundation Trust, London, UK; 4 Neurogenetics Unit, UCL Queen Square Institute of Neurology, London, UK; 5 Department of Clinical Neurophysiology, King’s College Hospital, London, UK; 6 Department of Clinical and Experimental Epilepsy, UCL Queen Square Institute of Neurology, London, UK; 7 Department of Pharmacology, UCL School of Pharmacy, London, UK

**Keywords:** skeletal muscle channelopathy, chloride channel, myotonia congenita, ClC-1, *CLCN1*

## Abstract

High-throughput DNA sequencing is increasingly employed to diagnose single gene neurological and neuromuscular disorders. Large volumes of data present new challenges in data interpretation and its useful translation into clinical and genetic counselling for families. Even when a plausible gene is identified with confidence, interpretation of the clinical significance and inheritance pattern of variants can be challenging. We report our approach to evaluating variants in the skeletal muscle chloride channel ClC-1 identified in 223 probands with myotonia congenita as an example of these challenges. Sequencing of *CLCN1*, the gene that encodes CLC-1, is central to the diagnosis of myotonia congenita. However, interpreting the pathogenicity and inheritance pattern of novel variants is notoriously difficult as both dominant and recessive mutations are reported throughout the channel sequence, ClC-1 structure-function is poorly understood and significant intra- and interfamilial variability in phenotype is reported.

Heterologous expression systems to study functional consequences of CIC-1 variants are widely reported to aid the assessment of pathogenicity and inheritance pattern. However, heterogeneity of reported analyses does not allow for the systematic correlation of available functional and genetic data. We report the systematic evaluation of 95 CIC-1 variants in 223 probands, the largest reported patient cohort, in which we apply standardized functional analyses and correlate this with clinical assessment and inheritance pattern. Such correlation is important to determine whether functional data improves the accuracy of variant interpretation and likely mode of inheritance.

Our data provide an evidence-based approach that functional characterization of ClC-1 variants improves clinical interpretation of their pathogenicity and inheritance pattern, and serve as reference for 34 previously unreported and 28 previously uncharacterized CLCN1 variants. In addition, we identify novel pathogenic mechanisms and find that variants that alter voltage dependence of activation cluster in the first half of the transmembrane domains and variants that yield no currents cluster in the second half of the transmembrane domain. None of the variants in the intracellular domains were associated with dominant functional features or dominant inheritance pattern of myotonia congenita.

Our data help provide an initial estimate of the anticipated inheritance pattern based on the location of a novel variant and shows that systematic functional characterization can significantly refine the assessment of risk of an associated inheritance pattern and consequently the clinical and genetic counselling.

## Teaser

Suetterlin *et al*. perform functional analyses of 95 missense variants of skeletal muscle chloride channel ClC-1, and correlate the data with mode of inheritance and clinical phenotype in 223 families with myotonia. The findings provide an evidence-based guide for improved genetic counselling of patients with myotonia.

## Introduction

Myotonia congenita is the most common skeletal muscle channelopathy.^1^ It is caused by a reduction in the repolarizing chloride current, resulting in an increase in the excitability of the muscle membrane,^2^ leading to a delay in terminating muscle contraction following voluntary activity. This manifests clinically as stiffness and rigidity of affected muscles. Myotonia congenita has dominant and recessive forms, both caused by mutations in *CLCN1* that result in reduced function of the encoded skeletal muscle chloride channel ClC-1.^3,4^ Sequencing of *CLCN1* has become integral to confirming myotonia congenita diagnosis following clinical assessment. However, it is important that myotonia is not erroneously attributed to an identified *CLCN1* variant as myotonia can also be caused by myotonic dystrophy (DM), a multisystem and potentially lethal disorder, and by gain-of-function mutations of the skeletal muscle sodium channel Na_v_1.4 (encoded by *SCN4A*). Mutations in distinct myotonia-associated genes (*CLCN1*, *SCN4A*, *DMPK*, *CNBP*) can co-occur in a patient and modify the presentation compared to a patient carrying a single gene mutation.^5-11^

ClC-1 is a homodimer.^12,13^ Each subunit contains its own chloride-selective pore and is composed of 18 intramembrane α-helices (conventionally numbered A to R) organized in two topologically related repeats with opposite membrane orientations, and an intracellular domain with two cystathionine beta-synthase (CBS) repeats. The chloride-selective pores can be gated individually or concurrently, in processes known as fast and slow gating, respectively. Both gates are opened by membrane depolarization. ClC-1 voltage sensitivity arises from the interaction of the channel with chloride ions.^14–16^

Interpretation of the clinical significance of a ClC-1 variant can be difficult as myotonia congenita exhibits intra- and interfamilial variability in the phenotype, severity and penetrance. In addition, it is currently difficult to accurately predict pathogenicity or inheritance of a novel variant based purely on the amino acid change and its location in the ClC-1 channel.

Functional assessment of mutations associated with myotonia congenita has revealed that they either reduce functional expression or shift the voltage dependence of channel activation towards depolarized voltages, thereby reducing the chloride current at physiological voltages. A mutant subunit can show dominant negative effects on coexpression with wild-type subunits, typically by shifting the voltage dependence of activation of the heterodimeric channel to depolarized voltages.^17,18^ Variants that show dominant negative effects in functional expression analysis are typically associated with dominant inheritance of myotonia congenita^4,17,18^ while variants without dominant negative effects are associated with recessive inheritance.^19–21^

To enable accurate use of functional data in a clinical diagnostic setting a correlation between distinct functional and clinical features needs to be established. This is currently complicated by phenotypic variability but also by heterogeneity in the methodology of the acquisition of genetic, clinical and functional data that often do not allow direct comparison of distinct functional features with the inheritance data between studies. In addition, some variants are found in both dominant and recessive pedigrees, suggesting that the correlation between functional features and the inheritance pattern is not linear. Finally, in some cases the functional data do not match the predicted pathogenicity and inheritance data.^22–24^ Thus, an evidence-based guide for assessment of pathogenicity and inheritance pattern based on specific functional features of ClC-1 variants is needed for purposeful clinical and genetic counselling.

We use functional expression to inform the diagnosis and genetic counselling in patients with myotonia congenita. Here we report analysis of the correlation of the functional properties of 95 distinct missense variants with the reported inheritance pattern of 223 probands in a diagnostic service setting and assess the implications for the use of functional data to improve genetic counselling in myotonia congenita.

## Materials and methods

### Ethics

The study was conducted as part of a service evaluation of the NHS England Highly Specialised Muscle Channelopathy Service at the National Hospital for Neurology and Neurosurgery. No procedures were performed outside of routine clinical care. The oocytes were recovered from *Xenopus laevis* toads in accordance with the Animals (Scientific Procedures) Act 1986.

### Genetics

The diagnostic molecular genetics laboratory at the National Hospital for Neurology and Neurosurgery is the UK national centre for myotonia congenita genetic testing. Since 2007 this consists of sequencing all 23 exons of *CLCN1* plus flanking intronic regions, or targeted sequencing of specific exons for relatives of individuals in whom variants have already been identified. For samples processed before 2007, it was routine for initial sequencing to be of mutation hotspots only. At least the proband subsequently underwent full sequencing of all *CLCN1* exons apart from the few circumstances where it was not possible to obtain DNA to do so. In cases where no mutations are found, a single recessive mutation is identified or a homozygous mutation is identified, Multiplex Ligation-dependent Probe Amplification is performed to assess for exon deletions or duplications. Whenever possible, the inheritance and allelic distribution of the variants is investigated by sequencing *CLCN1* in family members. Sequencing of *SCN4A* (full gene or hotspots), DM1 and DM2 retest was triggered after a ClC-1 variant was identified if the phenotype was atypical or it was uncertain if the *CLCN1* variant could account for dominant myotonia congenita. In some samples *SCN4A* and *CLCN1* were screened in parallel.

DNA was extracted from blood using standard methods. Bidirectional direct DNA sequencing was performed using a Big Dye Terminator sequencing kit [Applied Biosystems (ABI)] and a 3730 automated DNA sequencer (ABI). DNA sequences were analysed using v.2.5 SeqScape Analysis software (ABI). All 23 *CLCN1* exons are compared to larger databases including 1000 genomes, dbSNP, ExAC and Exome Variant Server.

### Clinical and genetic assessment of clinical inheritance pattern

The *CLCN1* variants in each pedigree were assigned as dominant, recessive or sporadic, based on available demographic, clinical, electrophysiological and genetic data that were collected from referral forms and/or clinic notes (Tables 1, 2 and Supplementary Tables 1–3). Dominant variants were single variants sufficient to cause the myotonia congenita phenotype and associated with parent to child transmission. The inheritance pattern of some dominant variants was specified ‘with variable penetrance’ (i) when the proband’s parents self-reported as asymptomatic but one was found to have the variant and clinical or electrographic myotonia on examination; or (ii) in families with no known history of consanguinity, parents self-reported as asymptomatic and a nephew, niece, aunt, uncle or half-sibling was reported to be affected. Recessive variants were found in homozygosis or compound heterozygosis and associated with asymptomatic parents. Sporadic variants were found in isolation in probands with asymptomatic parents and no other family history of myotonia congenita.

**Table 1:** Inheritance patterns of variants with wild-type-like or recessive functional features

Variant	Functional	Clinical/Genetic					Sum	Location
		Dominant	Sporadic	Recessive	Uncertain	Unknown		
p.His29Pro	Wild-type-like	–	–	1	–	–	1	IC
p.Ser70Leu	Wild-type-like	–	–	1	–	–	1	IC
p.Arg105Cys	Wild-type-like	–	–	–	1	–	1	IC
p.Leu106Val*	Wild-type-like	–	–	–	1	–	1	IC
p.Gln154Arg	Wild-type-like	–	–	–	1	–	1	TM1
p.Gly222Ser	Wild-type-like	–	–	–	1	–	1	TM1
p.Val327Ile	Wild-type-like	–	–	–	–	2	2	TM1
p.Ala331Ser*	Wild-type-like	–	–	1	–	–	1	TM1
p.Phe333Leu*	Wild-type-like	–	–	1	–	–	1	TM1
p.Ala402Val	Wild-type-like	–	1	1	–	–	2	TM2
p.Pro408Ala	Wild-type-like	–	–	2	1	3	6	TM2
p.Val456Ile*	Wild-type-like	–	–	–	–	1	1	TM2
p.Ala493Thr*	Wild-type-like	–	–	–	–	1	1	TM2
p.Phe494Leu*	Wild-type-like	–	–	–	1	–	1	TM2
p.Leu587Val*	Wild-type-like	–	–	2	–	–	2	IC
p.Gly594Val*	Wild-type-like	–	–	–	–	1	1	IC
p.Arg611His*	Wild-type-like	–	–	–	1	1	2	IC
p.Met646Thr*	Wild-type-like	–	–	1	–	–	1	IC
p.His664Pro*	Wild-type-like	–	–	–	1	–	1	IC
p.Arg669Cys	Wild-type-like	–	1	–	–	–	1	IC
p.Gly688Arg*	Wild-type-like	–	–	–	–	1	1	IC
p.Pro744Thr	Wild-type-like	–	–	–	1	–	1	IC
p.Thr837Ile*	Wild-type-like	–	–	–	1	–	1	IC
p.Val851Met	Wild-type-like	–	–	2	–	−.	2	IC
p.Gly898Arg*	Wild-type-like	–	–	–	1	–	1	IC
**Total**	**Wild-type-like**	**-**	**2**	**12**	**11**	**10**	**35**	
p.Phe167Leu	Recessive^a^	–	–	7	4	2	13	TM1
p.Gly190Arg	Recessive^b^	–	–	3	1	–	4	TM1
p.Leu198Val	Recessive^a^	–	–	–	1	–	1	TM1
p.Ala221Glu*	Recessive^b^	–	–	1	–	–	1	TM1
p.Gly233Ser	Recessive^b^	–	–	1	–	–	1	TM1
p.Val273Met	Recessive^a^	–	–	–	–	1	1	TM1
p.Gly276Ser	Recessive^a^	–	–	1	–	–	1	TM1
p.Cys277Arg	Recessive^b^	–	–	1	–	–	1	TM1
p.Gly285Val*	Recessive^b^	1	–	–	–	–	1	TM1
p.Glu291Lys	Recessive^b^	–	–	1	–	–	1	TM1
p.Arg317Leu	Recessive^a^	–	–	1	–	–	1	TM1
p.Ala320Val	Recessive^a^	–	–	1	–	–	1	TM1
p.Arg338Gln	Recessive^a^	–	–	1	–	1	2	TM1
p.Gly355Arg	Recessive^b^	–	–	1	–	–	1	TM2
p.His369Pro	Recessive^b^	–	–	–	–	1	1	TM2
p.Val397Asp*	Recessive^b^	–	–	–	–	1	1	TM2
p.Phe413Cys	Recessive^b^	–	–	–	–	1	1	TM2
p.Glu422Lys	Recessive^b^	–	–	–	1	–	1	TM2
p.Trp433Arg	Recessive^b^	–	–	–	–	1	1	TM2
p.Phe463Ile*	Recessive^b^	–	–	1	–	–	1	TM2
p.Gly483Ser*	Recessive^a^	–	–	1	–	–	1	TM2
p.Met485Val	Recessive^b^	–	–	6	4	1	11	TM2
p.Ala493Glu	Recessive^b^	–	–	1	–	–	1	TM2
p.Glu500Lys*	Recessive^b^	–	–	1	–	–	1	TM2
p.Pro521Thr*	Recessive^b^	–	–	1	–	–	1	TM2
p.Ala529Val*	Recessive^b^	–	–	–	–	1	1	TM2
p.Glu548Lys	Recessive^b^	–	–	–	–	1	1	TM2
p.Thr550Met	Recessive^b^	–	–	–	1	–	1	TM2
p.Pro558Ser	Recessive^b^	–	–	2	–	–	2	TM2
p.Met560Thr	Recessive^a^	–	–	–	–	1	1	TM2
p.Ala566Thr	Recessive^b^	–	–	2	–	–	2	TM2
p.Gln583Arg*	Recessive^a^	–	–	1	–	1	2	TM2
p.Val640Phe*	Recessive^b^	–	–	1	–	–	1	IC
p.Asp822Asn*	Recessive^a^	–	–	1	–	–	1	IC
p.Pro883Thr	Recessive^a^	–	–	1	–	2	3	IC
**Total**	**Recessive**	**1**	**-**	**38**	**12**	**15**	**66**	

Asterisks following variant name indicate variants not reported in the literature previously. Functional = classification according to functional feature; Clinical/Genetic = number of pedigrees with distinct inheritance patterns of clinical symptoms. Location = if the variant is found in intracellular domain (IC: residues 1–110, 586–988), first (TM1: residues 111–344) or second (TM2: residues 345–585) of the transmembrane repeats. Data for the variants that were not missense are presented in Supplementary Table 3.

a,b For recessive variants it is specified if the variant in homomeric condition expresses currents with shifted voltage dependence of activation^a^ or reduced current amplitude^b^.

**Table 2:** Inheritance patterns of variants with dominant or extraordinary functional features

Variant	Functional	Clinical/Genetic					Sum	Location
		Dominant	Sporadic	Recessive	Uncertain	Unknown		
p.Met128Ile	Dominant	2	–	–	–	–	2	TM1
p.Cys179Tyr*	Dominant	1	–	–	–	–	1	TM1
p.Ser183Pro	Dominant	1	–	–	–	–	1	TM1
p.Gly190Ser	Dominant	–	–	3	–	–	3	TM1
p.Leu198Pro	Dominant	–	–	–	–	1	1	TM1
p.Gly200Glu*	Dominant	–	–	1	–	–	1	TM1
p.Ala218Val	Dominant	1	–	–	–	–	1	TM1
p.Gly230Glu	Dominant	29	5	–	1	5	40	TM1
p.Pro234Thr*	Dominant	–	–	1	–	–	1	TM1
p.Pro234Leu*	Dominant	–	–	–	1	–	1	TM1
p.Thr268Met	Dominant	1	–	–	–	2	3	TM1
p.Cys271Arg	Dominant	1	–	–	–	–	1	TM1
p.Gly276Asp	Dominant	2	–	–	–	–	2	TM1
p.Gly285Glu	Dominant	5	3	9	4	5	26	TM1
p.Ser289Gly*	Dominant	1	–	–	–	–	1	TM1
p.Ser289Asn	Dominant	1	1	–	–	–	2	TM1
p.Ser289Ile*	Dominant	–	–	1	–	–	1	TM1
p.Phe297Ser	Dominant	7	–	–	–	–	7	TM1
p.Val299Leu	Dominant	–	–	2	–	–	2	TM1
p.Trp303Arg	Dominant	12	2	–	–	2	16	TM1
p.Phe306Leu	Dominant	2	1	–	–	2	5	TM1
p.Phe307Ser	Dominant	1	2	4	–	3	10	TM1
p.Ala313Thr	Dominant	9	1	1	–	1	12	TM1
p.Ala313Val	Dominant	2	–	–	2	1	5	TM1
p.Val321Leu	Dominant	–	1	–	–	–	1	TM1
p.Thr328Ile	Dominant	1	–	–	–	4	5	TM1
p.Pro480His	Dominant	–	–	–	2	–	2	TM2
p.Pro480Ser	Dominant	1	–	–	–	1	2	TM2
p.Gly523Asp	Dominant	–	1	–	–	–	1	TM2
p.Val536Ile	Dominant	–	–	–	–	1	1	TM2
p.Gly551Asp	Dominant	–	–	–	–	1	1	TM2
**Total**	**Dominant**	**80**	**17**	**22**	**10**	**29**	**158**	
p.Leu332Arg*	Other	–	1	–	–	–	1	TM1
p.Pro342Leu*	Other	–	1	–	–	–	1	TM1
p.Arg421Cys	Other	–	–	1	–	–	1	TM2
p.Met485Lys*	Other	–	–	1	–	–	1	TM2
**Total**	**All**	**81**	**21**	**74**	**33**	**54**	**263**	

Asterisks following variant name indicate variants not reported in the literature previously. Functional = classification according to functional feature; Clinical/Genetic = number of pedigrees with distinct inheritance patterns of clinical symptoms. Location = if the variant is found in intracellular domain (IC: residues 1–110, 586–988), first (TM1: residues 111–344) or second (TM2: residues 345–585) of the transmembrane repeats. ‘Other’ indicates that the functional features could not be classified as wild-type-like, dominant or recessive. Data in row ‘All’ includes data from Tables 1 and 2.

As expected at service level, the phenotyping and genotyping data for family members was not always complete. In Supplementary Tables 1–3 we specify whether the assessment of inheritance pattern was confirmed by segregation of the variant with the clinical symptoms or if it was based on reports. When a family history was not available or was insufficient to determine the inheritance pattern, the variant is listed as unknown (Tables 1, 2 and Supplementary Tables 1–3). Some variants were classified as ‘uncertain pathogenicity’ as specified in the ‘Results’ section.

### Molecular biology

The mutations were introduced into wild-type *CLCN1* cDNA by Quikchange site-directed mutagenesis (Agilent).^18^ Successful mutagenesis was confirmed by sequencing the entire insert. The mRNA was transcribed from MluI linearized vector using mMessageMachine SP6 kit (Ambion).

### *Xenopus laevis* oocytes

Oocytes were extracted from adult female *Xenopus laevis*,^25^ isolated after incubation with 2 mg/ml Collagenase Type A (Roche) in OR-2 (in mM): NaCl 82.5, KCl 2, MgCl_2_ 1, HEPES and injected with 2.5 ng of wild-type or mutant mRNA. The heterozygous condition is simulated by injecting a 1:1 mixture of wild-type and mutant mRNA. The oocytes were incubated in modified Barth’s solution (in mM): NaCl 88, KCl 1, MgSO_4_ 1.68, HEPES 10, Ca(NO_3_)_2_ 0.47, NaHCO_3_ 2.4, CaCl_2_ 0.41, supplemented with penicillin and streptomycin routinely for 30–72 h at ∼15°C before electrophysiological recordings. Each variant was studied in more than one batch of oocytes.

### Electrophysiology

Two-electrode voltage clamp experiments were performed using GeneClamp 500B, DigiData 1200 or 1550 Interface and Clampex software (all Axon Instruments) at room temperature in ND96 extracellular media (in mM): NaCl 96, KCl 2, MgCl_2_ 1, HEPES 5, CaCl_2_ 1.8, pH 7.4. Recording electrodes were filled with 3 M KCl and had a tip resistance <1 MΩ. Data were filtered at 1 kHz and sampled at 5 kHz.

From a holding voltage of −80 mV, an activating pre-pulse step to +60 mV for 250 ms was applied before test voltage steps ranging from −150 to +190 mV in 10 mV increments for 250 ms, followed by a tail voltage step to −100 mV. For most cells the same protocol was also applied with a holding voltage of −40 mV as well as an additional protocol where the holding voltage was −80 mV and the pre-pulse step taken to −140 mV. These protocols are referred to as *V*_h_ = −40mV and *V*_pp_ = −140 mV, respectively.

### Data analysis

Data analysis and presentation were prepared using Clampfit (Axon instruments), Origin (OriginLab) and Excel (Microsoft) software.

The tail currents were routinely measured 4 ms after the test pulse. The current–voltage relationship was fitted with the Boltzmann equation:

(1) $$I\left(V\right)=I_{\text{max}}/\left\{1+\text{exp}\;\left[\left(V_{1/2}-V\right)/V_{c}\right)]+C\right\}$$

where *I*_max_ is the amplitude of the fit, *C* the offset current, *V*_1/2_ the voltage at which the current is (*I*_max_ + *C*) / 2 and *V*_c_ the slope factor. We fixed the value of *C* in the fitting process to the baseline current level at the most hyperpolarized voltages. In some cases, two Boltzmann equations were required to fit the data:

(2) $$I\left(V\right)=C+I_{\text{max}}*(A/\left\{1+\text{exp}\;\;[\left(V_{1/2(1)}-V\right)/V_{c1})]\right\}+\left(1-A\right)/\left\{1+\text{exp}\;\left[\left(V_{1/2(2)}-V\right)/V_{c2}\right]\right\}\}$$

with *C* and *I*_max_ as before while *A*, *V*_1/2(1),_*V*_c1_ and (1 − *A*), *V*_1/2(2),_*V*_c2_ are the fraction, the voltage of half-maximal activation and the slope factor of the first and second Boltzmann component, respectively. The time constant of activation was assessed by fitting a two-component exponential equation to data following settling of capacitive transients, and the weighted average of time constants is presented.

Cells without a clear component of activation that could be described with a Boltzmann equation and with tail current amplitude <1 µA following a step to +80 mV were considered devoid of functional ClC-1 channel expression.

Data are presented as mean ± standard error of the mean (SEM) unless otherwise stated. To assess if clustering of the variants across the functional and structural groups was significant, we used a two-tailed Fisher’s test.

### Data availability

Functional data are available on reasonable request to the corresponding author. The clinical and genetic data are not publicly available.

## Results

### Genetic and clinical overview of the cohort

Our cohort comprised 223 probands referred for genetic testing for myotonia congenita in whom *CLCN1* variants were identified. A total of 115 distinct mutations were identified in the cohort, 95 of which were missense while 20 were non-missense (truncating, frameshifting, intronic, duplications, deletions or silent). The non-missense variants were included in the cohort only if they were compound heterozygous with missense mutations.

A single heterozygous variant was found in 116, homozygous variant in 27 (one with two homozygous variants), compound heterozygous variants in 75 and more than two variants in five probands (Supplementary Table 1). In total, 309 variants (Supplementary Tables 1–3), of which 263 were missense (Supplementary Table 2), were found in the cohort and were assigned an inheritance pattern. For the missense variants this was either dominant (81), sporadic (21) or recessive (74) (Supplementary Table 2). For a further 54 missense variants, family history was unavailable or insufficient to determine the inheritance pattern. For 33 missense variants the assessment of pathogenicity or inheritance was complicated by co-allelic *CLCN1* variants, the presence of variants in other myotonia-associated genes, by the variant not segregating with myotonia congenita symptoms, or by reported dominant inheritance in compound heterozygous probands where it could not be determined which of the variants was associated with dominant inheritance. These variants were assigned ‘with uncertain pathogenicity’. For 50 of the missense variants in the cohort with recessive or dominant inheritance patterns, the assessment was based on both clinical and genetic segregation data and for 105 variants this was based on clinical symptoms only (Supplementary Table 2). Of the 33 pedigrees with dominant inheritance and confirmed segregation, six met our criteria for variable penetrance (Supplementary Table 2).

Seventy-eight of the variants were found in a single pedigree only (64 missense variants) (Supplementary Tables 1–3). The most common variant, G230E, was identified in 40 pedigrees. Eight variants were found associated with more than one inheritance pattern (dominant, sporadic, recessive), of which three (G285E, F307S and A313T) were associated with both recessive and dominant inheritance. The only variant with genetic segregation data available to confirm association with both dominant and recessive inheritance was G285E. Variable penetrance has been reported previously for G285E.^18,19,26^

To our knowledge, 34 of the missense variants have not been previously reported as associated with myotonia congenita and a further 28 have been reported but not functionally characterized.

### Functional assessment and classification of ClC-1 variants

**Figure 1:**
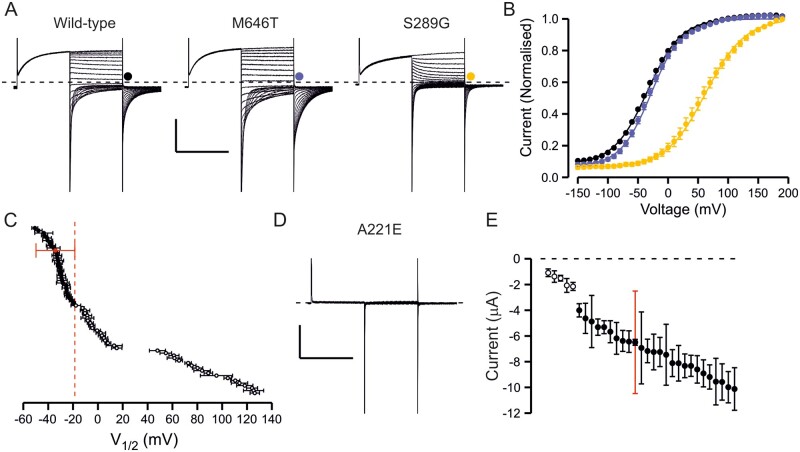
**Overview of functional properties of ClC-1 variants.** (**A**) Representative traces of wild-type channel, a variant with wild-type-like functional features (M646T) and a variant with shifted voltage dependence of activation (S289G). From holding voltage of −80 mV, 250 ms pre-pulse step to +60 mV, 250 ms test voltage steps from −150 mV to +190 mV in 10-mV increments (only traces in response to pulses up to +60 mV are shown) and a tail voltage step to −100 mV were applied. Scale bars: = 250 ms (*x*), 3 µA (*y*). (**B**) Voltage dependence of activation of wild-type (black), M646T (blue) and S289G (yellow) channels. Current at the beginning of tail voltage step was normalized to peak amplitude of the Boltzmann fit for each cell and the mean ± SEM normalized current data are shown. Solid lines show fit of the Boltzmann equation to mean data. (**C**) Mean *V*_1/2_ ± SEM is shown for each variant. If the *V*_1/2_ of the variant was depolarized relative to the cut-off voltage (wild-type ± 1.5× SD red dashed vertical line) the variant was considered pathogenic. If the *V*_1/2_ was to the left of the cut-off it was considered wild-type-like. (**D**) Representative current traces of a variant (A221E) with minimal ClC-1 currents. Scale bars as in **A**. (**E**) Mean ± SEM tail current amplitude of variants with wild-type-like voltage dependence. Red bars show SD of wild-type current amplitude data. While most variants showed wild-type-like current amplitude, for five variants many cells did not express currents and when the currents were detectable the mean amplitude was outside the limits of wild-type ± SD. These variants were considered pathogenic due to reduced expression. Numbers are shown in Supplementary Table 4.

The effect of the *CLCN1* missense variants on ClC-1 channel function was tested in the *Xenopus laevis* oocyte expression system for the 95 missense variants. The *V*_1/2_ for wild-type channels was −34.2 ± 0.6 mV (*n* = 308) (Fig. 1 and Supplementary Table 4). As the *V*_1/2_ of cells expressing wild-type channels was variable [standard deviation (SD) = 10.4 mV, range −68.6 to −7.8 mV], we assigned a cut-off voltage (*V*_1/2_ for the wild-type channel ±1.5× SD = −18.6 mV) to decide whether the *V*_1/2_ was wild-type-like or pathogenic (Fig. 1C). Using this cut-off voltage, the well-known pathogenic variant F167L with a modest positive shift in the voltage dependence of activation^27^ (*V*_1/2_ = −17.7 mV; Supplementary Table 4) was classified as pathogenic while variants with V_1/2_ negative to that were not. One variant, H664P, activated at more hyperpolarized voltages than 1.5× SD cut-off (Supplementary Table 4). In the absence of loss-of-function effects this variant was not considered pathogenic.

The current amplitude of wild-type channels was also variable (−6.3 ± 0.2 µA, SD = 3.6 µA, range −0.5 to −30 µA). Twenty-five variants did not show any chloride currents (Supplementary Table 4) and for five variants with wild-type-like voltage dependence of activation (A221E, H369P, V397D, F413C, E422K) and one variant with shifted voltage dependence of activation (W303R) many of the oocytes did not show currents (Fig. 1 and Supplementary Table 4) and the mean amplitude of the currents from those oocytes that yielded currents was less than the mean + SD for oocytes expressing wild-type channels (Fig. 1E). Although not displaying complete loss-of-function these variants were considered pathogenic by reducing functional expression of the channel.

Most (91/95) of the channel variants could be described as having wild-type-like features (25 variants), no or reduced chloride currents (31 variants) or chloride currents with shifted voltage dependence of activation (35 variants) (Fig. 1 and Supplementary Table 4).

### Functional properties of extraordinary ClC-1 variants

Four variants showed properties that could not be described by reduced current amplitude or shifted voltage dependence of activation alone.

**Figure 2:**
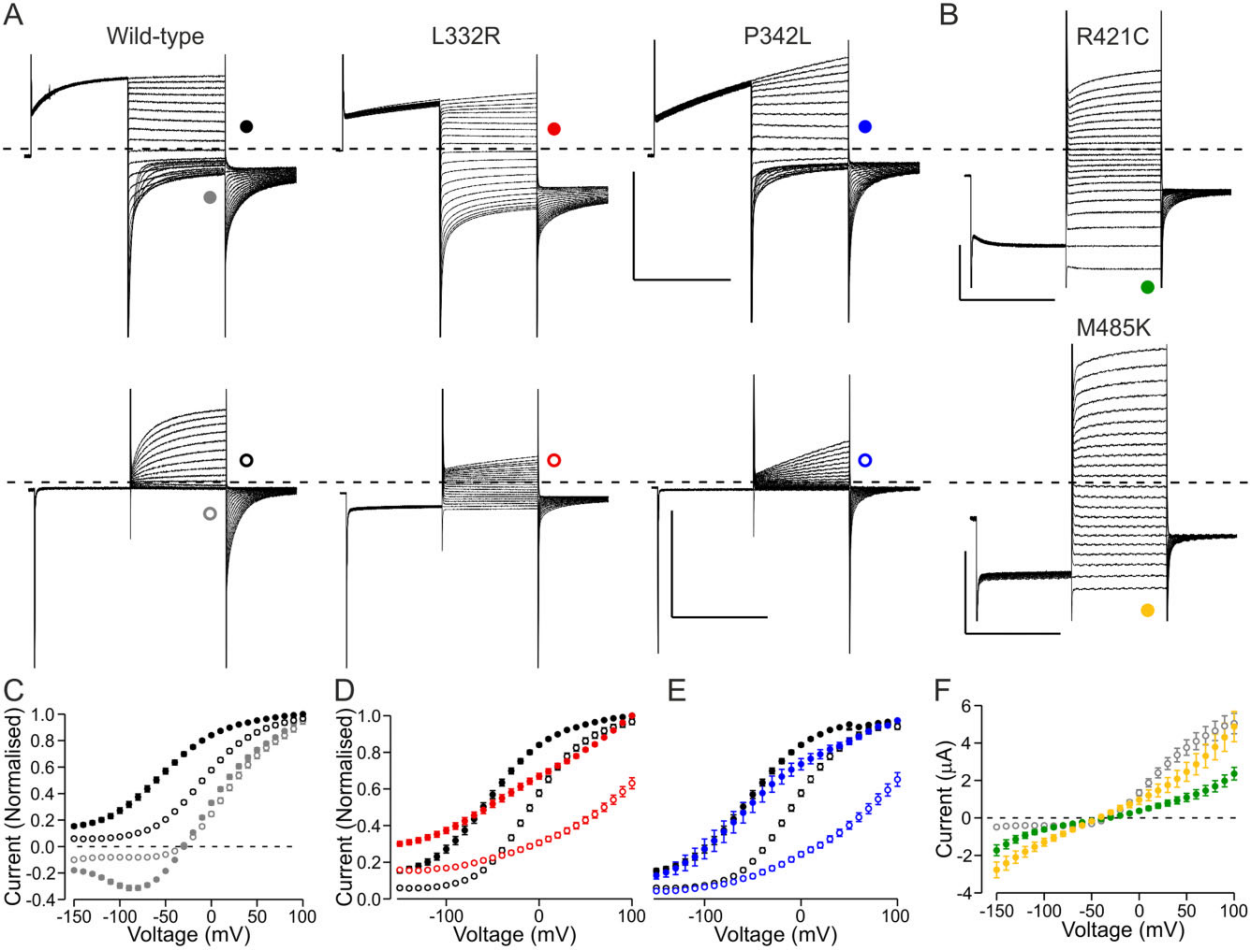
**Functional properties of variants where the dysfunction could not be described by analysis of *V*_1/2_ or current amplitude only.** (**A**) Representative current traces of wild-type, L332R and P342L channels. Voltage protocol was as in Fig. 1A except that the holding voltage was −40 mV (*V*_h_ = −40 mV) (*top row*) or as in Fig. 1A but the pre-pulse step was to −140 mV (*V*_PP_ = −140 mV) (*bottom row*). Scale bars = 250 ms (*x*), 5 µA (*y*). (**B**) Representative current traces of R421C and M485K channels. Voltage protocol was as in Fig. 1A except that the pre-pulse step was to −140 mV. (**A** and **B**) Traces in response to test pulses up to +60 mV are shown. Scale bars = 250 ms (*x*), 1 µA (R421C) or 3 µA (M485K) (*y*). (**C**) Data for wild-type channels are shown in response to test voltages (grey) or to tail voltage (black) using the *V*_h_ = −40 mV protocol (solid symbols) and *V*_PP_ = −140 mV protocol (open symbols). Data for each cell are normalized to current in response to (grey) or immediately after (black) a voltage step to +100 mV. Only a subset of wild-type cells was analysed (*n* = 18). (**D** and **E**) Data for L332R (red) and P342L (blue) are shown for *V*_h_ = −40 mV (solid) and *V*_PP_ = −140 mV (open) protocols. Data are normalized as in **C**. L332R *n* = 13 and P342L *n* = 11. Wild-type data as in **C** are shown in black. (**F**) Mean current at the end of the test pulse is plotted against the test voltage for R421C (green) (*n* = 19) and M485K (*n* = 9) channels. Wild-type data for cells in **C** are shown in grey.

Two variants (L332R and P342L) showed depolarization-activated currents but it was difficult to describe the voltage dependence of activation with a Boltzmann equation. In addition, the tail current amplitude declined when studied with pre-pulses to hyperpolarized voltages (*V*_PP_ = −140 mV) but increased when using a more depolarized holding voltage (*V*_h_ = −40mV) (Fig. 2A, D and E). The voltage dependence of activation of wild-type channels too is clearly dependent on the voltage protocol (Fig. 2A and C). To compare wild-type, L332R and P342L behaviours, the currents for each cell were normalized to the tail current amplitude following a voltage step to +100 mV using the *V*_h_ = −40 mV protocol. In response to a voltage step to 0 mV using the *V*_PP_ = −140 mV protocol the normalized wild-type-channel activity was 58%, but only 31% for L332R and 24% for P342L channels, demonstrating reduced activity for mutant channels at physiological voltages (Fig. 2D and E).

Two variants (M485K and R421C) showed currents at hyperpolarized voltages (Fig. 2B and F). M485K channels also showed depolarization-activated currents but with a voltage dependence of activation that was shifted to depolarized voltages (Supplementary Table 4). Oocytes expressing R421C channels showed small depolarization-activated currents (mean tail current amplitude −1.1 ± 0.1 µA) with shifted voltage dependence of activation (Supplementary Table 4). The current amplitude at −120 mV was −1.8 ± 0.3 µA for M485K cells, −0.9 ± 0.2 µA for R421C channels but −0.4 ± 0.05 µA for wild-type cells (*V*_pp_ = −140 mV protocol).

### Assessment of dominant negative effect of ClC-1 variants

**Figure 3:**
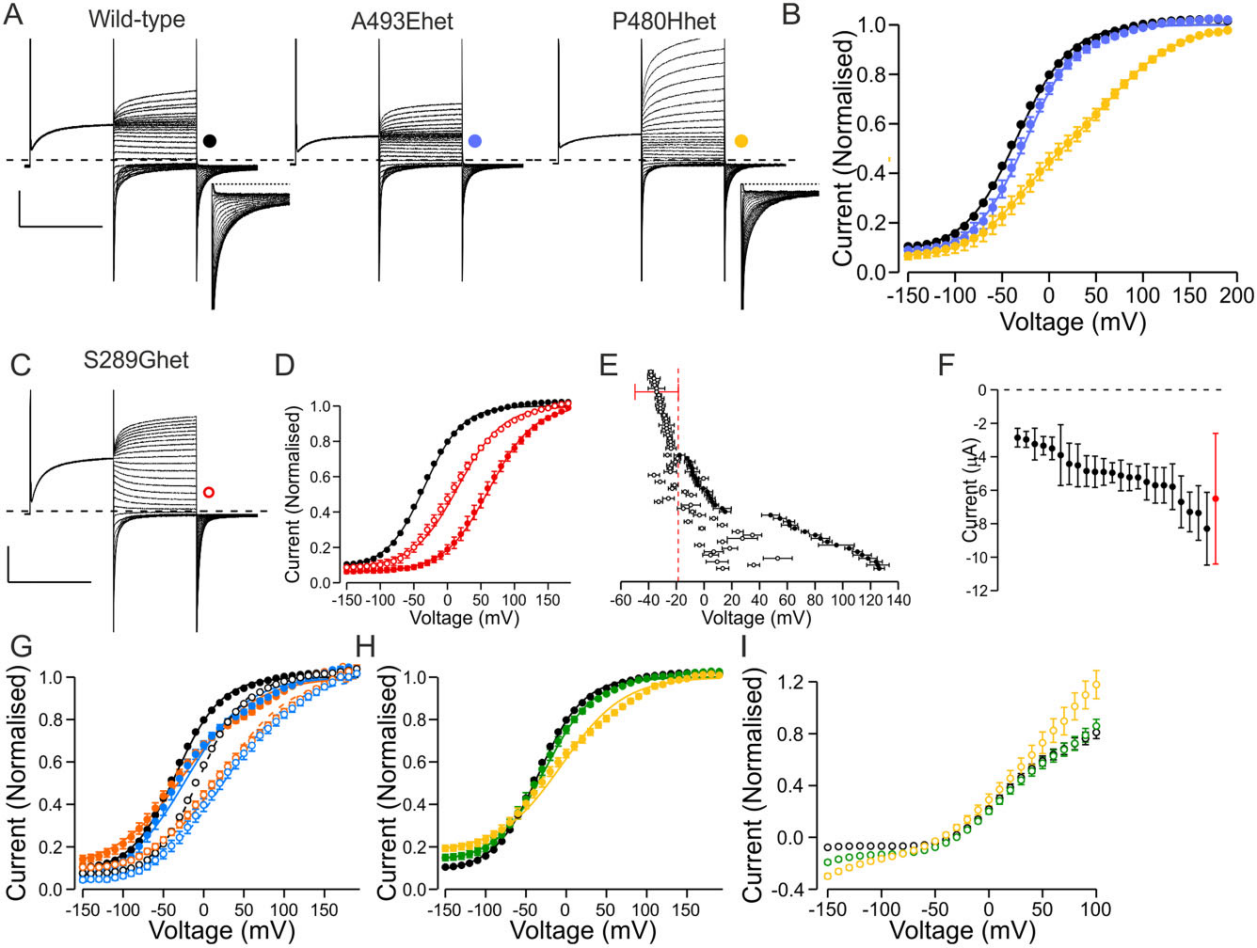
**Properties of simulated heterozygous mutant channels.** (**A**) Representative current traces of wild-type channels and mutant channels that displayed no currents in the homomeric condition. In simulated heterozygous condition these either showed currents with wild-type-like (A493Ehet) or shifted (P480Hhet) voltage dependence of activation. Inserts show a zoom in on the first 90 ms of the tail current to illustrate two distinct components of activation for P480Hhet channels. Scale bars = 250 ms (*x*), 3 µA (*y*). (**B**) Voltage dependence of activation of wild-type, A493Ehet and P480Hhet channels. The data were normalized as in Fig. 1B except for P480Hhet where tail currents in individual cells were normalized to peak amplitude of a double Boltzmann equation. (**C**) Representative current traces of a variant with shifted voltage dependence of activation in homomeric condition (S289G) co-expressed with wild-type subunits. Scale bars = 250 ms (*x*), 3 µA (*y*). (**D**) Voltage dependence of activation of wild-type (black), S289G (solid red) and S289Ghet (open red) channels. (**E**) *V*_1/2_ of simulated heterozygous channels. Mean wild-type data ± 1.5× SD cut-off voltage is shown in red, data in homomeric conditions with solid symbols and in simulated heterozygous conditions with open symbols. For variants with no, or reduced currents, in homomeric conditions only data in simulated heterozygous condition are shown. Variants with *V*_1/2_ left of the cut-off voltage were considered recessive, while variants right of the cut-off voltage were considered dominant. Data for variants for which two-component Boltzmann equation was used to describe the voltage dependence are not included. (**F**) Current amplitude of simulated heterozygous variants that in homomeric condition showed no or reduced currents and in heterozygous conditions showed wild-type-like voltage dependence. None of the variants suppressed current amplitude to below 50% of wild-type current amplitude, consistent with an absence of dominant negative effects on wild-type subunit function. (**G**) Voltage dependence of activation of L332Rhet (orange) and P342Lhet (blue) channels using the *V*_h_ = −40 mV (solid symbols) or *V*_PP_ = −140 mV (open symbols) protocols. While the *V*_1/2_ was wild-type-like using the *V*_h_ = −40 mV protocol, this was shifted when using the *V*_PP_ = −140 mV protocol [*V*_1/2_(wild-type) = −4.4 ± 0.4 mV, *n* = 271, *V*_1/2_(L332Rhet) = 27.0 ± 4.1 mV, *n* = 8, *V*_1/2_(P342L) = 28.4 ± 4.9 mV, *n* = 6]. (**H**) Voltage dependence of activation of R421Chet (green) and M485Khet (yellow) channels. (**I**) Voltage dependence of currents at the end of the test voltage pulse for R421Chet (green) and M485Khet (yellow) measured using the *V*_PP_ = −140 mV protocol. Data were normalized to peak tail current amplitude of the same cell measured using the standard protocol. The normalized current data indicate increased currents at hyperpolarized voltages for the simulated heterozygous mutant channels compared to wild-type channels.

To simulate the heterozygous condition of the patient and to assess the inheritance pattern of the variant, mRNA encoding mutant and wild-type subunits were co-injected into oocytes (Fig. 3). We indicate the simulated heterozygous condition by adding the suffix ‘het’ to the variant name, e.g. F167Lhet.

Twenty-three of the 31 variants that showed no or reduced currents when expressed alone showed currents with wild-type-like voltage dependence of activation in simulated heterozygous conditions (Fig. 3E and Supplementary Table 4). The current amplitude in simulated heterozygous condition was not reduced much below 50% of current amplitude from oocytes expressing wild-type channels (Fig. 3F).

Eight variants with no or reduced currents as homomers produced currents with altered voltage sensitivity of activation on coexpression (Fig. 3B and Supplementary Table 4). The voltage dependence of the heterozygous channels was better fit with Boltzmann equation with two rather than with one component. The component that activated at more hyperpolarized voltages had a *V*_1/2_ similar to that of wild-type channels (Supplementary Table 4), consistent with these currents being produced by a mixed population of wild-type homomers and wild-type/mutant dimers.

For all variants with a shift in the voltage dependence of activation, the *V*_1/2_ of the simulated heterozygous channel was less positive than that of the homomeric mutant channel, except for the F297S variant that showed a greater shift in the *V*_1/2_ in simulated heterozygous than in homomeric form (Fig. 3E and Supplementary Table 4). For 23 variants, the *V*_1/2_ was positive to the voltage set as a cut-off for assigning pathogenicity in homomeric conditions. This suggests that in heterozygous conditions the current amplitude is significantly reduced at physiological voltages. For 12 variants, the voltage dependence in simulated heterozygous conditions was wild-type-like.

Thus, in total 35 variants that showed pathogenic changes in homomeric condition did not show dominant negative effects on channel function in simulated heterozygous condition suggesting recessive inheritance, while 31 variants showed a shifted voltage dependence of activation, suggesting dominant inheritance.

The voltage dependence of activation of P342Lhet and L332Rhet channels could be fitted with a Boltzmann equation and was wild-type-like (Fig. 3 and Supplementary Table 4). However, when using the *V*_pp_ = −140 mV protocol the voltage dependence of activation was shifted about +30 mV compared to wild-type, suggesting that the simulated heterozygous form retains an increased sensitivity to voltage protocols (Fig. 3). The current amplitude at hyperpolarized voltages relative to the amplitude at depolarized voltages was larger for M485Khet and R421Chet channels compared to wild-type channels (Fig. 3). The voltage dependence of the depolarization-activated current was shifted for M485Khet and wild-type-like for R421Chet channels (Supplementary Table 4).

### Correlation of functional properties with inheritance patterns of clinical symptoms

We next analysed how the distinct functional features of the 95 missense variants (‘Functional’ column Tables 1 and 2 and Supplementary Table 4) correlates with the inheritance pattern assigned by analysing the clinical and genetic features of the 223 probands (‘Clinical/Genetic’ column in Tables 1, 2 and Supplementary Table 2). To ensure equal weight for each variant, the variants that were associated with more than one inheritance category were assigned a fractional value based on the frequency they appeared in the distinct categories (‘Clinical/Genetic’ column in Tables 1 and 2). For example, A313T was found in 12 probands: nine dominant, one sporadic and one recessive pedigree, and in one pedigree the inheritance was unknown. It was assigned with 0.75 dominant, 0.083 sporadic, 0.083 recessive and 0.083 unknown inheritance.

Wild-type-like functional features suggest the variant is not associated with myotonia congenita. Accordingly, the pathogenicity was uncertain for 39% of these variants and for a further 24% the data were insufficient to confirm the inheritance pattern. For only 37% of these variants was clinical data sufficient to assess the inheritance pattern: two probands (6%) were sporadic carrying a lone heterozygous variant with asymptomatic parents and the remaining variants (31%) were found in recessive pedigrees.

For variants with recessive functional features the family history was available to confirm recessive inheritance for 58%. For 28% the inheritance pattern could not be confirmed and for 11% the association with myotonia congenita was uncertain. All probands with unknown inheritance pattern and all but one of the probands with uncertain association with myotonia congenita carried compound heterozygous *CLCN1* variants. Only two variants with recessive functional features were found as a lone mutation. G285V was found in heterozygosis in a dominant pedigree with a parent carrying the variant and displaying mild myotonia. DM1, DM2 and *SCN4A* mutations were excluded in the proband. In another pedigree, the association of a lone F167L variant with myotonia congenita was uncertain as it was identified together with a known pathogenic *SCN4A* variant.

The non-missense variants were compound heterozygous with missense variants as per inclusion criteria. Consistently, when the inheritance pattern of clinical symptoms could be determined it was always recessive (Supplementary Table 3). Pathogenicity of some of the non-missense variants included in this cohort remains to be determined.

For missense variants with dominant functional features the inheritance pattern was dominant for 41%, sporadic for 11%, recessive for 19%, unknown for 21% and uncertain for 8% of variants. For 8/10 pedigrees where the association with myotonia congenita was uncertain the inheritance was reported dominant but the proband carried two *CLCN1* variants and it could not be confirmed which variant was dominant. Two other uncertain cases include probands where a heterozygous A313V or G285E variant occurred together with a known pathogenic *SCN4A* variant.

Two of the variants with a mild positive shift in *V*_1/2_ in the simulated heterozygous condition compared to the cut-off voltage, A313T and P480S (Supplementary Table 4), were identified mainly in dominant pedigrees (75 and 50%, respectively). In contrast, all variants with shifted voltage dependence of activation in the homomeric condition but wild-type-like voltage dependence in the simulated heterozygous condition were identified in recessive pedigrees. This suggests that the cut-off *V*_1/2_ value was useful in discerning variants associated with recessive inheritance from variants with a risk of dominant inheritance.

**Figure 4:**
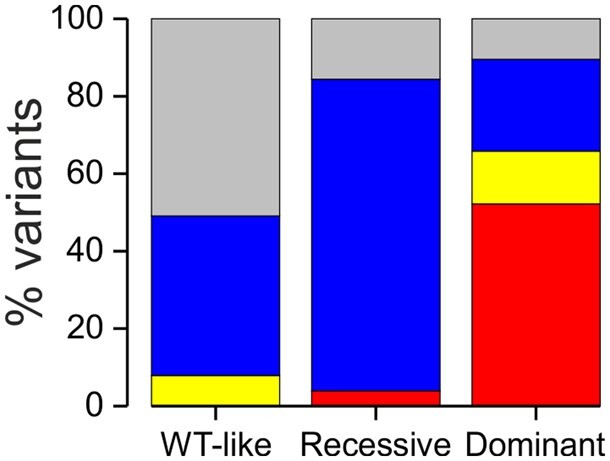
**Correlation of the functional properties with the inheritance pattern of clinical symptoms.** Bar graph of inheritance patterns of all pedigrees for missense variants with wild-type-like (WT-like), recessive and dominant functional features. Dominant inheritance pattern is indicated in red, sporadic inheritance in yellow and recessive inheritance in blue. Variants with uncertain association with clinical symptoms are shown in grey. Variants with unknown inheritance pattern are excluded from the figure.

For statistical analysis of the correlation of functional and inheritance data, we excluded the variants with insufficient information to determine the inheritance pattern (Fig. 4). The percentage of variants with uncertain association with myotonia congenita was significantly higher for variants with wild-type-like functional features (51%) than variants with recessive (16%, *P* < 0.05) or dominant (10%, *P* < 0.01) functional features. The percentage of variants with recessive inheritance pattern of clinical symptoms was significantly higher for variants with recessive functional features (80%) than variants with wild-type-like (41%, *P* < 0.01) or dominant (24%, *P* < 0.001) functional features. The percentage of variants associated with a dominant inheritance pattern of clinical symptoms was significantly higher for variants with dominant functional features (52%) than variants with wild-type-like (0%, *P* < 0.001) or recessive (4%, *P* < 0.001) functional features. Sporadic variants showed dominant or wild-type-like but not recessive functional features.

Variants with prominent loss-of-function following a hyperpolarizing pre-pulse (L332R, P342L) were found in pedigrees with sporadic inheritance, suggesting that a single variant may be sufficient to cause clinical symptoms. The M485K variant with hyperpolarization-activated currents and shifted voltage dependence of depolarization-activated currents in the simulated heterozygous condition was found in a large pedigree with recessive inheritance. The inheritance pattern for R421C with hyperpolarization-activated currents and wild-type-like depolarization-activated currents in the simulated heterozygous condition was also recessive, with an asymptomatic heterozygous carrier among the parents. F297S, the only variant with *V*_1/2_ more positive in the simulated heterozygous than in the homomeric condition, was only found in dominant pedigrees.

### Alternative pathogenic mechanisms of variants with wild-type-like voltage dependence of activation

More than a quarter of the studied variants show wild-type-like expression levels and voltage dependence of activation, despite being found in patients with clinical features suggestive of myotonia congenita. Accordingly, for many of these variants the association with myotonia was uncertain, but in several pedigrees the clinical and genetic data support an association with myotonia congenita. We investigated whether functional features other than current amplitude or voltage dependence of activation could indicate alternative pathogenic mechanisms.

**Figure 5:**
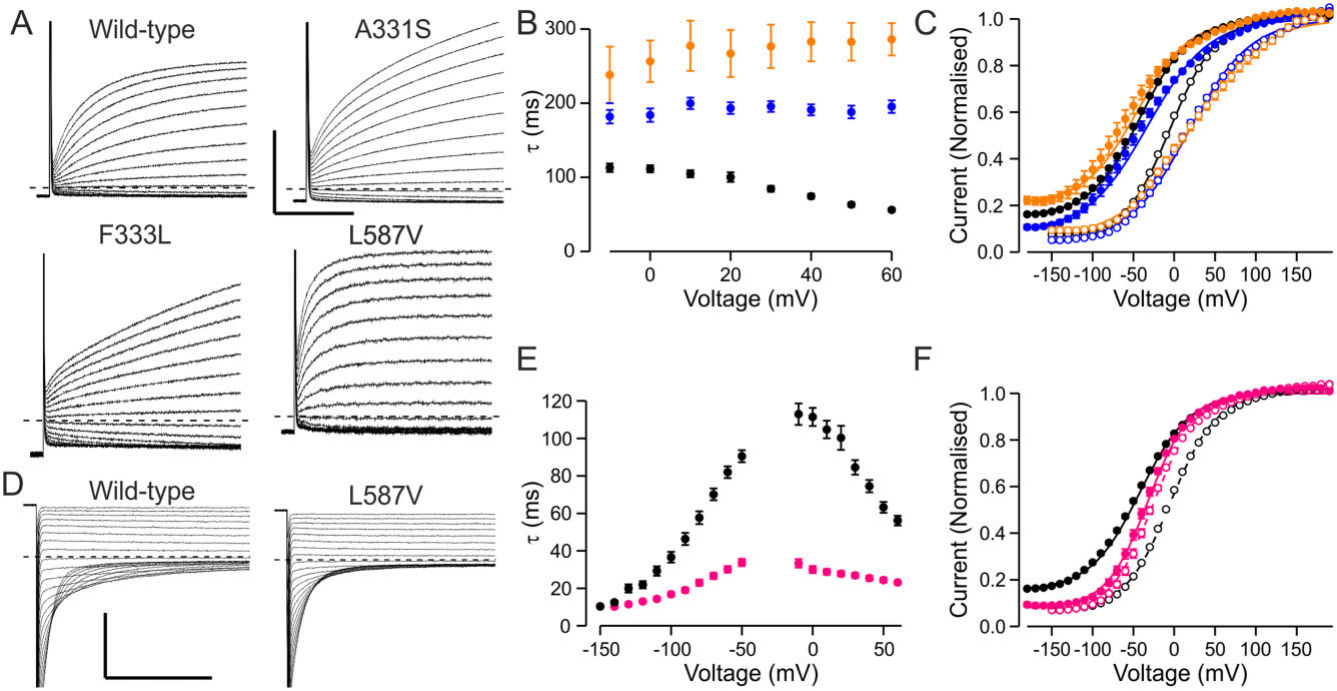
**Alternative pathogenic mechanisms.** (**A**) Representative current traces showing time course of activation of wild-type, A331S, F333L and L587V. Holding voltage was −80 mV, responses steps to voltages between −60 and +60 mV are shown. (**A** and **D**) Scale bars = 50 ms (*x*), 5 µA (*y*). (**B** and **C**) Time constant (**B**) and voltage dependence (**C**) of activation for wild-type (black), A331S (blue) and F333L (orange) channel. Solid symbols show data for *V*_h_ = −40 mV protocol, open symbols for *V*_PP_ = −140 mV protocol. (**D**) Representative current traces showing time course of deactivation of wild-type and L587V channels. Holding voltage was −80 mV, traces show the time course of closure following pre-pulse to +60 mV for voltage range +50 to −150 mV. (**E** and **F**). Time constants of activation and deactivation (**E**) and voltage dependence of activation (**F**) for wild-type (black) and L587V (pink) channels. Solid symbols show data for the *V*_h_ = −40 mV protocol, open symbols for the *V*_PP_ = −140 mV protocol.

Two variants, A331S and F333L, showed a wild-type-like voltage dependence of activation following a depolarizing pre-pulse (Supplementary Table 4). However, both showed clearly reduced rates of activation (Fig. 5A and B). Consequently, following a hyperpolarizing pre-pulse (*V*_pp_ = −140 mV), the voltage dependence of activation was shifted about +25 mV compared to wild-type channels (Fig. 5C). A331S was found in a proband with P480fs frameshift mutation with asymptomatic parents while F333L was found in homozygosis in a pedigree where the parents were confirmed heterozygous asymptomatic carriers. These data indicate that slow activation may contribute to myotonia congenita with recessive inheritance.

One variant with wild-type-like voltage dependence of activation, L587V, showed an accelerated rate of activation and closing (Fig. 5A, D and E). The variant was found in homozygosis in two pedigrees with confirmed asymptomatic parents, suggesting that the variant contributes to recessive myotonia congenita. Accelerated closing is a loss-of-function feature that results in reduced increment in current amplitude at physiological voltages when switching from hyperpolarizing to depolarizing pre-pulse conditions. For wild-type channels the mean open probability at −60 mV increases from 0.17 to 0.47 (276%) when switching from hyperpolarizing (*V*_pp_ = −140mV protocol) to depolarizing (*V*_h_ = −40 mV protocol) pre-pulse condition. Respective values for L587V are 0.24 (*V*_pp_ = −140 mV) and 0.31 (*V*_h_ = −40 mV) [29% increase, *P* < 0.001 for both protocols (Mann–Whitney test)]. These data indicate that a lack of increase in ClC-1 channel activity at physiological voltage following electrical activation of the muscle may contribute towards myotonia.

## Discussion

Our functional data for 95 ClC-1 missense variants identified in 223 myotonic probands provide strong support for an evidence-based guide for the use of functional data in the diagnosis of myotonia congenita. Thirty-four of these ClC-1 variants were novel and 28 had not previously been functionally characterized, some of which showed new pathogenic mechanisms that have implications for understanding the function of the CLC-1 channel. We also identified clustering of variants with distinct functional features on the ClC-1 structure.

### Variants with wild-type-like functional features

Consistent with the lack of detrimental effects in functional analysis, variants with wild-type-like functional features are significantly more likely to have uncertain association with myotonia congenita compared to variants with pathogenic functional features. However, an association with myotonia congenita is not excluded when a variant is functionally wild-type-like based on voltage dependence and current amplitude alone. Other loss-of-function features may contribute, such as alterations in rates of channel activation or deactivation (Fig. 5) or splicing, as shown for V327Lvariant.^28^ Another variant c.1222A>G (P408A) creates an AG dinucleotide that activates a cryptic acceptor site and potentially alters splicing of *CLCN1* mRNA (Human Splicing Finder). Potential splicing defects could be assessed by studying patient *CLCN1* mRNA or with the minigene assay.^29^ It is also possible that some variants with wild-type-like functional features but classified as recessive (H29P and S70V) are in fact innocuous as they were compound heterozygous with variants with dominant functional features (P234T and F307S, respectively). The classification as recessive may be a result of variable penetrance of the dominant variants, as shown previously for F307S variant. Finally, it is possible that the pathogenic mechanism involves disruption of muscle-specific modulation of the channel that cannot be detected in the *Xenopus* oocyte expression system. ClC-1 modulation involves intracellular domains^30,31^ where many of the variants with wild-type-like functional features are located (Table 1, see below).

When a variant with wild-type-like functional feature was associated with myotonia congenita the inheritance pattern was never dominant (Fig. 4). However, two variants with wild-type-like functional features (A402V and R669C) were found as a lone mutation in sporadic pedigrees with asymptomatic parents. It remains to be determined whether and how these variants with no detectable pathogenicity in functional assay are the sole cause of myotonia in these probands.

Taken together, our data indicate that variants with wild-type-like functional properties carry a significant risk of uncertain association with myotonia congenita and consequently alternative genetic causes of myotonia, e.g. *SCN4A* mutations and myotonic dystrophy should be excluded as a priority, particularly when a wild-type-like variant is found in isolation.

### Variants with recessive functional features

Variants with recessive functional features were mainly associated with recessive myotonia congenita (Fig. 4). In keeping with this, all probands with functionally recessive variants for whom the clinical inheritance pattern was unknown had compound heterozygous variants. Only one variant (G285V) was found in isolation in a pedigree with dominant family history. The mechanism of dominant inheritance of this variant remains to be determined.

These data suggest that variants with recessive functional properties should be reported as recessive and that finding these variants as the sole heterozygous variant should trigger investigations into alternative mechanisms of myotonia.

### Variants with dominant functional features

Fifty-two per cent of variants with dominant functional features were found in pedigrees with dominant inheritance of clinical symptoms (Fig. 4B), 14% in heterozygous probands with sporadic inheritance of myotonia congenita and a further 10% in dominant pedigrees although classified with ‘uncertain association with myotonia congenita’ as they were compound heterozygous with another *CLCN1* variant and segregation data were not available to confirm which variant was associated with dominant inheritance or with a known pathogenic *SCN4A* variant. It is likely that the *CLCN1* variants modify the presentation of *SCN4A* variants in these probands.^8–11^

The clinical inheritance of 24% of the functionally dominant variants was recessive. Although for most of these variants the shift in the voltage dependence of activation was modest, S289I, a variant with one of the largest shifts was found in a recessive pedigree with a reportedly asymptomatic parent. Dominant inheritance had variable penetrance in 7% of variants, including the most common dominant variant G230E that was found in two apparently unaffected individuals. In our cohort variants with dominant functional features had greatly increased risk of dominant inheritance compared to those with recessive or wild-type-like functional features. However, it is possible that none of the variants with dominant functional features has full clinical penetrance.

### Structure-function considerations

**Figure 6:**
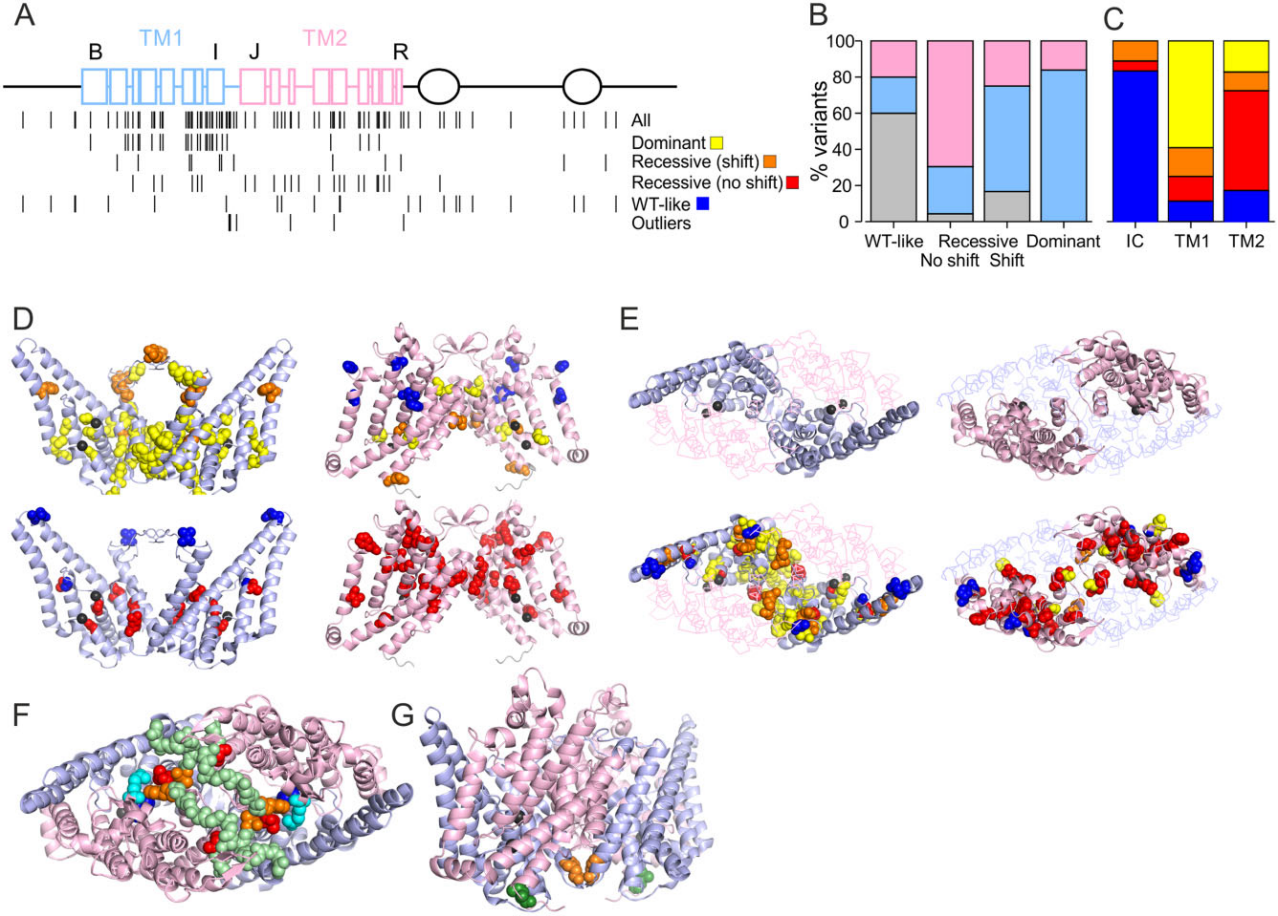
**Mapping of substituted residues (variants) to ClC-1 structure.** (**A**) Two-dimensional map of variants. *Top*: ClC-1 intramembrane helices are represented as squares, CBS domains as ovals and the connecting loops as lines. Blue indicates TM1, pink TM2, black intracellular (IC). Helices B, I, J and R are indicated. *Bottom*: Location of all the variants in this cohort is shown on the *top row* and location of the variants with distinct functional features is specified below. Two rows of recessive variants are shown depending on if the variant in homomeric condition shifted voltage dependence [Recessive (shift)] or just reduced functional expression [Recessive (no shift)]. Outliers include the variants shown in Figs 2 and 5. (**B**) Percentage of variants located in intracellular domains (grey), TM1 (blue) or TM2 (pink) is plotted for variants with distinct functional features. (**C**) Percentage of variants with dominant (yellow), recessive (shift) (orange) or recessive (no shift) (red) and wild-type-like functional features (blue) are plotted for variants located in the intracellular domains (IC), TM1 or TM2. (**D**) Mapping variants to TM1s (*left*, light blue) and TM2 (*right*, light pink) to ClC-1 structure (6COY).^22^ TMs of both subunits are shown, variants are plotted on both subunits, Cl^−^ are shown in black. View is on membrane plane. Cl^−^ ions (black) on left and right graphs are aligned to illustrate location of TM2 higher up in membrane plane compared to TM1. T*op row* shows dominant variants in yellow and Recessive (shift) in orange. *Bottom row* shows Recessive (no shift) variants in red. Variants with wild-type-like functional features are shown in blue. (**E**) *Top row* shows a view from above membrane plane with the two TM1s (*left*, light blue) or TM2s (*right*, light pink) shown in a cartoon while the other TM is shown in ribbon. On the *bottom row* all variants are mapped based on their functional group as in **B**. Most of subunit interface is formed by the two TM1s (*top*) and most variants that shift voltage dependence of activation at any condition are located here. (**F**) Variants with attenuated activation particularly following hyperpolarized pre-pulse (L332 and P342 in red, A331 and F333 in orange) are shown viewed from above the membrane plane. These variants are located on IJ-linker (main chain is shown in light green spheres) that forms an interface with the IJ-linker of the neighbouring subunit and reaches the proximity of variants that showed enhanced currents at hyperpolarized voltage [R421C (magenta) and M485K (cyan)]. (**G**) Location of L587V (green) variant that accelerated both opening and closing of the channel at the intracellular opening of the selectivity filter pathway. The F297S (orange) variant that displayed a larger shift in voltage dependence of activation when co-expressed with wild-type subunits compared to homomeric F297S channels is also shown. Note that the variants mentioned in **F** or the L587V variant are not shown in **B** or **C**.

When plotted on the ClC-1 primary structure a disproportionately high number of variants (48%) were located in the first of the roughly identical halves of the transmembrane domain (TM1) of ClC-1 subunit (helices B-I and the IJ-linker, residues 111–344) but only 19% of variants were found in the intracellular N and C termini (1–110, 586–988) (Tables 1, 2 and Fig. 6). Seventy-nine per cent (37/47) of the variants that shifted the voltage dependence of activation in homomeric or simulated heterozygous condition, including the variants with extraordinary functional properties, are located in TM1, while a further eight (17%) were on the second half of transmembrane domain (TM2) (residues 345–585) and two variants (4%) were intracellular (Fig. 6A–C). Of the variants with shifted voltage dependence of activation in simulated heterozygous condition 84% (26/31) were found on TM1 and the remaining 16% on TM2. In contrast, 16/23 (70%) of the variants with recessive functional features without shift in voltage dependence of activation in homomeric condition were found on TM2, six in TM1 (26%) and a single variant was intracellular. Finally, wild-type-like variants were predominantly found in intracellular termini (60%, 15/25), while five variants were found in TM1 and TM2 each. This distinct, predominant distribution of variants with dominant, recessive without shift in voltage dependence of activation as homomers and wild-type-like functional features in TM1, TM2 and intracellular domains, respectively, was significant (Fig. 6B, C) and helps guide preliminary predictions on functional features and consequently on pathogenicity and inheritance pattern of novel variants. However, functional characterization is still necessary as different substitutions of a single residue can have drastically different effects (e.g. M485V and M485K). Nevertheless, it is notable that in our cohort none of the variants found in the intracellular domains were associated with dominant inheritance or functional features. Therefore, in patients with a lone intracellular ClC-1 variant, alternative mechanisms of myotonia should be excluded as a priority.

TM1 is mainly located towards the intracellular end of the intramembrane domain where the chloride ions are found in the selectivity filter pathway (Fig. 6D).^32^ The voltage dependence of ClC-1 channel activation is sensitive to Cl^−^ concentration and arises from interactions with the channel and the ion.^14–16^ It is thus likely that the dominant variants localized towards the intracellular side shift the voltage dependence of activation by altering these interactions. The TM1 also forms most of the subunit interface (Fig. 6E), suggesting that mutations in one subunit can affect the chloride conducting pathway in the neighbouring subunit through this interface. F297S, the only variant with voltage dependence of activation showing a larger shift in simulated heterozygous than in the homomeric condition, affects a residue located at the subunit interface with the main chain of the two F297 residues in close contact (Fig. 6G). Functional data indicate that a symmetric F297S channel shows a smaller shift in voltage dependence of channel activation than a F297/F297S heterodimer, and supports the notion that the functional effect of a mutation in one subunit is physically transduced to the second subunit through the subunit interface (Fig. 6G).

TM2 is located mainly towards the extracellular side of the intramembrane domain, above the selectivity filter pathway. This may explain the lower frequency of variants that shift the voltage dependence of activation. The variants with shifted voltage dependence in TM2 are located close to the Cl^−^ ions (P480, G483, V536) or in the proximity of the TM1 of the neighbouring subunit (G523, G551, M560) (Fig. 6D). TM2 also contains a cluster of recessive variants without shift in voltage dependence of activation in homomeric condition located above the selectivity filter (Fig. 6D). It is not known whether these variants reduce channel expression or if they prevent the permeation of chloride by obstructing the pathway. Absence of significant subunit interface between two TM2s may explain why the effect of a mutation in one subunit is not commonly transduced to the neighbouring subunit.

Mutations that showed currents at hyperpolarized voltages affect the residues R421 and M485 located just above the glutamate residue (E232) implicated as an external gate where other mutations with similar gating defects are found (Fig. 6F).^33^ In particular, R421 forms a salt bridge with D136 that when replaced by a glycine conducts hyperpolarization^33^ activated currents. These data imply that residues above the selectivity filter form a gate that prevents hyperpolarization-activated currents.

The wild-type ClC-1 channels exhibit a low activity state (*V*_1/2_ ∼ −5 mV) induced by hyperpolarization and a high activity state (*V*_1/2_ ∼ −45 mV) induced by depolarization, thereby displaying hysteresis in its voltage dependence. Transition from a low to high activity state would allow for larger increase of ClC-1 currents at physiological voltages in response to prolonged muscle activity compared to a model where ClC-1 channel activity follows a constant voltage dependence. The notion that some mutants (L332R and P342L) showed more pronounced defects in response to hyperpolarizing pre-conditions suggests that the properties of the mutant channels should be assessed using both high and low activity protocols. This idea is reinforced by the observation that two mutant channels (A331S and F333L) with a reduced rate of activation when measured using hyperpolarized pre-pulses showed wild-type-like voltage dependence of activation when studied with protocols that incorporated a depolarizing pre-pulse. The unusual voltage dependence of activation of L332R and P342L channels following hyperpolarization may be a result of ultraslow activation (Fig. 2). All of these variants are found in the linker that connects helices I (TM1) and J (TM2) at the extracellular surface of the channel. The IJ-linkers of the two subunits are in close contact but the tip of the linker (L332/F333L) reaches the proximity of residues important for channel gating (R421) (Fig. 6F). Our data indicate that the IJ-linker is important for determining the rate of channel activation.

One of the variants in our cohort (L587V) showed a reduced shift between low to high activity states, a feature that can be attributed to accelerated channel opening and closing. L587V was found in homozygosis in two myotonia congenita pedigrees suggesting that the distinct kinetic features contribute towards myotonia and that a normal shift from low to high activity state on electrical activation of the muscle may be important for preventing myotonia. The slow component of wild-type channel closure may be a result of ([Cl^−^]-dependent) stabilization of the open state that shifts the voltage dependence of activation to more hyperpolarized voltages. The location of L587 suggests that conformational changes that stabilize the open state occur at the intracellular entrance of the selectivity filter pathway (Fig. 6G).

### Implications for clinical practice

One aim of this work was to improve the genetic counselling that we can provide to patients. The first application of the data is that even without functional characterization, an initial approximate, but evidence-based estimate of the risk of an associated dominant inheritance pattern can be drawn based on the location of the variant (Fig. 6A). In particular, variants with dominant functional features are clustered in TM1 and variants with recessive functional features without shift in voltage dependence of activation are clustered in TM2. None of the variants in intracellular domains in our cohort were associated with dominant family history or dominant functional features suggesting that for heterozygous variants in intracellular domains other causes of myotonia should be excluded as a priority. The location-based estimate can be refined by closer investigation of the location in the 3D structure of the ClC-1 channel (Fig. 6).^32,34^ Second, once functional characterization is performed, this risk of an associated dominant inheritance pattern can be significantly refined (Fig 4).

Functional assessment of pathogenicity and inheritance for 95 ClC-1 variants found in 223 myotonia congenita pedigrees shows a clear correlation between recessive and dominant functional features with respective inheritance patterns of clinical symptoms. It provides strong support and an evidence-based guide for the use of functional analysis in the genetic diagnosis and counselling of myotonia congenita. Accurate diagnostic and genetic counselling may help guide the therapeutic strategies of patients with myotonia congenita.^35^

We believe our evidence-based guide is translatable to laboratories using the same protocols for functional analysis. Laboratories using different protocols will need to perform a correlation analysis between functional features and the inheritance pattern of clinical symptoms to establish their own criteria to classify the functional features. The data provided in this paper serve as a reference for the inheritance pattern of 95 variants to help establish such correlations.

## Funding

The work was supported by University College London Hospitals National Institute for Health Research Biomedical Research Centre and by the UK Medical Research Council. K.S. was supported by a Medical Research Council Clinical Research Training Fellowship (MR/M01827X/1) and by U FP7/2007–2013 programme grant no. 2012–305121 ‘NeurOmics’. E.M. received funding from a Wellcome Clinical Research Career Development Fellowship (209583/Z/17/Z).

## Competing interests

Authors report no competing interests.

## Supplementary material

Supplementary material is available at *Brain* online.

## Supplementary Material

awab344_Supplementary_Data

## References

[1] Horga A , Raja Rayan DL, Matthews E, et al Prevalence study of genetically defined skeletal muscle channelopathies in England. Neurology. 2013;80(16):1472–1475. 23516313 10.1212/WNL.0b013e31828cf8d0 PMC3662361

[2] Bryant SH , Morales-Aguilera A. Chloride conductance in normal and myotonic muscle fibres and the action of monocarboxylic aromatic acids. J Physiol. 1971;219(2):367–383. 5316641 10.1113/jphysiol.1971.sp009667 PMC1331636

[3] Koch MC , Steinmeyer K, Lorenz C, et al The skeletal muscle chloride channel in dominant and recessive human myotonia. Science (New York, NY). 1992;257(5071):797–800. 10.1126/science.1379744 1379744

[4] George AL Jr , Crackower MA, Abdalla JA, Hudson AJ, Ebers GC. Molecular basis of Thomsen's disease (autosomal dominant myotonia congenita). Nat Genet. 1993;3(4):305–310. 7981750 10.1038/ng0493-305

[5] Bugiardini E , Rivolta I, Binda A, et al *SCN4A* mutation as modifying factor of myotonic dystrophy type 2 phenotype. Neuromuscul Disord. 2015;25(4):301–307. 25660391 10.1016/j.nmd.2015.01.006

[6] Binda A , Renna LV, Bosè F, et al *SCN4A* as modifier gene in patients with myotonic dystrophy type 2. Sci Rep. 2018;8(1):11058. 30038349 10.1038/s41598-018-29302-z PMC6056531

[7] Cardani R , Giagnacovo M, Botta A, et al Co-segregation of DM2 with a recessive *CLCN1* mutation in juvenile onset of myotonic dystrophy type 2. J Neurol. 2012;259(10):2090–2099. 22407275 10.1007/s00415-012-6462-1

[8] Furby A , Vicart S, Camdessanche JP, et al Heterozygous *CLCN1* mutations can modulate phenotype in sodium channel myotonia. Neuromuscul Disord. 2014;24(11):953–959. 25088311 10.1016/j.nmd.2014.06.439

[9] Kato H , Kokunai Y, Dalle C, et al A case of non-dystrophic myotonia with concomitant mutations in the *SCN4A* and *CLCN1* genes. J Neurol Sci. 2016;369:254–258. 27653901 10.1016/j.jns.2016.08.030

[10] Maggi L , Ravaglia S, Farinato A, et al Coexistence of *CLCN1* and *SCN4A* mutations in one family suffering from myotonia. Neurogenetics. 2017;18(4):219–225. 28993909 10.1007/s10048-017-0525-5

[11] Thor MG , Vivekanandam V, Sampedro-Castañeda M, et al Myotonia in a patient with a mutation in an S4 arginine residue associated with hypokalaemic periodic paralysis and a concomitant synonymous *CLCN1* mutation. Sci Rep. 2019;9(1):17560. 31772215 10.1038/s41598-019-54041-0 PMC6879752

[12] Miller C , White MM. Dimeric structure of single chloride channels from Torpedo electroplax. Proc Natl Acad Sci U S A. 1984;81(9):2772–2775. 6326143 10.1073/pnas.81.9.2772 PMC345152

[13] Dutzler R , Campbell EB, Cadene M, Chait BT, MacKinnon R. X-ray structure of a ClC chloride channel at 3.0 A reveals the molecular basis of anion selectivity. Nature. 2002;415(6869):287–294. 11796999 10.1038/415287a

[14] Richard EA , Miller C. Steady-state coupling of ion-channel conformations to a transmembrane ion gradient. Science (New York, NY). 1990;247(4947):1208–1210. 10.1126/science.2156338 2156338

[15] Pusch M , Ludewig U, Rehfeldt A, Jentsch TJ. Gating of the voltage-dependent chloride channel CIC-0 by the permeant anion. Nature. 1995;373(6514):527–531. 7845466 10.1038/373527a0

[16] Rychkov GY , Pusch M, Astill DS, Roberts ML, Jentsch TJ, Bretag AH. Concentration and pH dependence of skeletal muscle chloride channel ClC-1. J Physiol. 1996;497(Pt 2):423–435. 8961185 10.1113/jphysiol.1996.sp021778 PMC1160994

[17] Pusch M , Steinmeyer K, Koch MC, Jentsch TJ. Mutations in dominant human myotonia congenita drastically alter the voltage dependence of the CIC-1 chloride channel. Neuron. 1995;15(6):1455–1463. 8845168 10.1016/0896-6273(95)90023-3

[18] Fialho D , Schorge S, Pucovska U, et al Chloride channel myotonia: Exon 8 hot-spot for dominant-negative interactions. Brain. 2007;130(Pt 12):3265–3274. 17932099 10.1093/brain/awm248

[19] Kubisch C , Schmidt-Rose T, Fontaine B, Bretag AH, Jentsch TJ. ClC-1 chloride channel mutations in myotonia congenita: Variable penetrance of mutations shifting the voltage dependence. Hum Mol Genet. 1998;7(11):1753–1760. 9736777 10.1093/hmg/7.11.1753

[20] Mazon MJ , Barros F, De la PP, et al Screening for mutations in Spanish families with myotonia. Functional analysis of novel mutations in *CLCN1* gene. Neuromuscul Disord. 2012;22(3):231–243. 22094069 10.1016/j.nmd.2011.10.013

[21] Vindas-Smith R , Fiore M, Vasquez M, et al Identification and functional characterization of *CLCN1* mutations found in nondystrophic myotonia patients. Hum Mutat. 2016;37(1):74–83. 26510092 10.1002/humu.22916

[22] Wollnik B , Kubisch C, Steinmeyer K, Pusch M. Identification of functionally important regions of the muscular chloride channel CIC-1 by analysis of recessive and dominant myotonic mutations. Hum Mol Genet. 1997;6(5):805–811. 9158157 10.1093/hmg/6.5.805

[23] Imbrici P , Maggi L, Mangiatordi GF, et al ClC-1 mutations in myotonia congenita patients: Insights into molecular gating mechanisms and genotype-phenotype correlation. J Physiol. 2015;593(18):4181–4199. 26096614 10.1113/JP270358 PMC4594292

[24] Altamura C , Lucchiari S, Sahbani D, et al The analysis of myotonia congenita mutations discloses functional clusters of amino acids within the CBS2 domain and the C-terminal peptide of the ClC-1 channel. Hum Mutat. 2018;39(9):1273–1283. 29935101 10.1002/humu.23581

[25] Parrock S , Hussain S, Issler N, et al KCNJ10 mutations display differential sensitivity to heteromerisation with *KCNJ16*. Nephron Physiol. 2013;123(3-4):7–14. 24193250 10.1159/000356353

[26] Stunnenberg BC , Raaphorst J, Deenen JCW, et al Prevalence and mutation spectrum of skeletal muscle channelopathies in the Netherlands. Neuromuscul Disord. 2018;28(5):402–407. 29606556 10.1016/j.nmd.2018.03.006

[27] Zhang J , Bendahhou S, Sanguinetti MC, Ptácek LJ. Functional consequences of chloride channel gene (*CLCN1*) mutations causing myotonia congenita. Neurology. 2000;54(4):937–942. 10690989 10.1212/wnl.54.4.937

[28] Lorenz C , Meyer-Kleine C, Steinmeyer K, Koch MC, Jentsch TJ. Genomic organization of the human muscle chloride channel CIC-1 and analysis of novel mutations leading to Becker-type myotonia. Hum Mol Genet. 1994;3(6):941–946. 7951242 10.1093/hmg/3.6.941

[29] Ulzi G , Sansone VA, Magri F, et al In vitro analysis of splice site mutations in the *CLCN1* gene using the minigene assay. Mol Biol Rep. 2014;41(5):2865–2874. 24452722 10.1007/s11033-014-3142-5

[30] Bennetts B , Parker MW, Cromer BA. Inhibition of skeletal muscle ClC-1 chloride channels by low intracellular pH and ATP. J Biol Chem. 2007;282(45):32780–32791. 17693413 10.1074/jbc.M703259200

[31] Hsiao KM , Huang RY, Tang PH, Lin MJ. Functional study of CLC-1 mutants expressed in *Xenopus* oocytes reveals that a C-terminal region Thr891-Ser892-Thr893 is responsible for the effects of protein kinase C activator. Cell Physiol Biochem. 2010;25(6):687–694. 20511714 10.1159/000315088

[32] Park E , MacKinnon R. Structure of the CLC-1 chloride channel from *Homo sapiens*. eLife. 2018;7:e36629. 10.7554/eLife.36629 PMC6019066 29809153

[33] Fahlke C , Rüdel R, Mitrovic N, Zhou M, George AL. An aspartic acid residue important for voltage-dependent gating of human muscle chloride channels. Neuron. 1995;15(2):463–472. 7646898 10.1016/0896-6273(95)90050-0

[34] Wang K , Preisler SS, Zhang L, et al Structure of the human ClC-1 chloride channel. PLoS Biology. 2019;17(4):e3000218. 31022181 10.1371/journal.pbio.3000218 PMC6483157

[35] Desaphy JF , Altamura C, Vicart S, Fontaine B. Targeted therapies for skeletal muscle ion channelopathies: Systematic review and steps towards precision medicine. J Neuromuscul Dis. 2021;8(3):357–381. 33325393 10.3233/JND-200582 PMC8203248

