# Supplementary material for: Translating genetic and functional data into clinical practice: a series of 223 families with myotonia
Source: Brain. 2021 Sep 16;145(2):607–20. doi: 10.1093/brain/awab344 (PMC9014745; doi:10.1093/brain/awab344)
Supplement: awab344_Supplementary_Data [file awab344_supplementary_data.pdf]

Proband	Nucleotide	Protein	Dominant			Sp	Recessive		Unknown		Uncertain	
			SC	VP	SR		SC	SR	Het	CH	Het	CH
**Heterozygous**												
1	c.384G>A	p.Met128Ile	I									
2	c.384G>A	p.Met128Ile			I							
3	c.501C>G	p.Phe167Leu									I	
4	c.536G>A	p.Cys179Tyr			I							
5	c.593T>C	p.Leu198Pro							I			
6	c.653C>T	p.Ala218Val			I							
7	c.689G>A	p.Gly230Glu	I									
8	c.689G>A	p.Gly230Glu	I									
9	c.689G>A	p.Gly230Glu	I									
10	c.689G>A	p.Gly230Glu	I									
11	c.689G>A	p.Gly230Glu	I									
12	c.689G>A	p.Gly230Glu	I									
13	c.689G>A	p.Gly230Glu	I									
14	c.689G>A	p.Gly230Glu	I									
15	c.689G>A	p.Gly230Glu	I									
16	c.689G>A	p.Gly230Glu		I								
17	c.689G>A	p.Gly230Glu							I			
18	c.689G>A	p.Gly230Glu			I							
19	c.689G>A	p.Gly230Glu			I							
20	c.689G>A	p.Gly230Glu			I							
21	c.689G>A	p.Gly230Glu			I							
22	c.689G>A	p.Gly230Glu			I							
23	c.689G>A	p.Gly230Glu			I							
24	c.689G>A	p.Gly230Glu			I							
25	c.689G>A	p.Gly230Glu			I							
26	c.689G>A	p.Gly230Glu			I							
27	c.689G>A	p.Gly230Glu			I							
28	c.689G>A	p.Gly230Glu			I							
29	c.689G>A	p.Gly230Glu			I							
30	c.689G>A	p.Gly230Glu			I							
31	c.689G>A	p.Gly230Glu			I							
32	c.689G>A	p.Gly230Glu			I							
33	c.689G>A	p.Gly230Glu			I							
34	c.689G>A	p.Gly230Glu			I							
35	c.689G>A	p.Gly230Glu				I						
36	c.689G>A	p.Gly230Glu				I						
37	c.689G>A	p.Gly230Glu				I						
38	c.689G>A	p.Gly230Glu				I						
39	c.689G>A	p.Gly230Glu				I						
40	c.689G>A	p.Gly230Glu							I			
41	c.689G>A	p.Gly230Glu							I			
42	c.803C>T	p.Thr268Met							I			
43	c.811T>C	p.Cys271Arg	I									

44	c.827G>A	p.Gly276Asp	1						
45	c.827G>A	p.Gly276Asp	1						
46	c.854G>A	p.Gly285Glu							1
47	c.854G>A	p.Gly285Glu			1				
48	c.854G>A	p.Gly285Glu			1				
49	c.854G>A	p.Gly285Glu				1			
50	c.854G>A	p.Gly285Glu				1			
51	c.854G>A	p.Gly285Glu				1			
52	c.854G>A	p.Gly285Glu						1	
53	c.854G>T	p.Gly285Val	1						
54	c.866G>A	p.Ser289Asn			1				
55	c.866G>A	p.Ser289Asn				1			
56	c.890T>C	p.Phe297Ser	1						
57	c.890T>C	p.Phe297Ser	1						
58	c.890T>C	p.Phe297Ser			1				
59	c.890T>C	p.Phe297Ser			1				
60	c.890T>C	p.Phe297Ser			1				
61	c.890T>C	p.Phe297Ser			1				
62	c.890T>C	p.Phe297Ser			1				
63	c.907T>C	p.Trp303Arg	1						
64	c.907T>C	p.Trp303Arg	1						
65	c.907T>C	p.Trp303Arg						1	
66	c.907T>C	p.Trp303Arg			1				
67	c.907T>C	p.Trp303Arg			1				
68	c.907T>C	p.Trp303Arg			1				
69	c.907T>C	p.Trp303Arg			1				
70	c.907T>C	p.Trp303Arg			1				
71	c.907T>C	p.Trp303Arg			1				
72	c.907T>C	p.Trp303Arg			1				
73	c.907T>C	p.Trp303Arg			1				
74	c.907T>C	p.Trp303Arg			1				
75	c.907T>C	p.Trp303Arg				1			
76	c.907T>C	p.Trp303Arg				1			
77	c.916T>C	p.Phe306Leu			1				
78	c.916T>C	p.Phe306Leu		1					
79	c.916T>C	p.Phe306Leu				1			
80	c.916T>C	p.Phe306Leu						1	
81	c.916T>C	p.Phe306Leu						1	
82	c.920T>C	p.Phe307Ser			1				
83	c.920T>C	p.Phe307Ser				1			
84	c.920T>C	p.Phe307Ser				1			
85	c.920T>C	p.Phe307Ser						1	
86	c.920T>C	p.Phe307Ser						1	
87	c.937G>A	p.Ala313Thr	1						
88	c.937G>A	p.Ala313Thr	1						
89	c.937G>A	p.Ala313Thr	1						
90	c.937G>A	p.Ala313Thr			1				
91	c.937G>A	p.Ala313Thr			1				
92	c.937G>A	p.Ala313Thr			1				

93	c.937G>A	p.Ala313Thr			1						
94	c.937G>A	p.Ala313Thr			1						
95	c.937G>A	p.Ala313Thr			1						
96	c.937G>A	p.Ala313Thr				1					
97	c.937G>A	p.Ala313Thr							1		
98	c.938C>T	p.Ala313Val	1								
99	c.938C>T	p.Ala313Val									1
100	c.938C>T	p.Ala313Val			1						
101	c.961G>C	p.Val321Leu				1					
102	c.983C>T	p.Thr328Ile							1		
103	c.995T>G	p.Leu332Arg				1					
104	c.1025C>T	p.Pro342Leu				1					
105	c.1205C>T	p.Ala402Val				1					
106	c.1366G>A	p.Val456Ile							1		
107	c.1438C>T	p.Pro480Ser	1								
108	c.1568G>A	p.Gly523Asp				1					
109	c.1652G>A	p.Gly551Asp							1		
110	c.1781G>T	p.Gly594Val							1		
111	c.1832G>A	p.Arg611His									1
112	c.1832G>A	p.Arg611His							1		
113	c.2005C>T	p.Arg669Cys				1					
114	c.2062G>C	p.Gly688Arg							1		
115	c.2510C>T	p.Thr837Ile									1
116	c.2692G>A	p.Gly898Arg									1
**Homozygous**											
117	c.547T>C	p.Ser183Pro			1						
118	c.662C>A	p.Ala221Glu						1			
119	C.697G>A	p.Gly233Ser					1				
120	c.865A>G	p.Ser289Gly		1							
121	c.871G>A	p.Glu291Lys					1				
122	c.895G>C	p.Val299Leu						1			
123	c.895G>C	p.Val299Leu					1				
124	c.920T>C	p.Phe307Ser								1	
125	c.937G>A	p.Ala313Thr						1			
126	c.983C>T	p.Thr328Ile			1						
127	c.983C>T	p.Thr328Ile								1	
128	c.983C>T	p.Thr328Ile								1	
129	c.983C>T	p.Thr328Ile								1	
130	c.999C>G	p.Phe333Leu					1				
131	c.1190T>A	p.Val397Asp								1	
132	c.1454T>A	p.Met485Lys						1			
133	c.1498G>A	p.Glu500Lys						1			
134	c.1642G>A	p.Glu548Lys								1	
135	c.1696G>A	p.Ala566Thr					1				
136	c.1759C>G	p.Leu587Val						1			
137	c.1759C>G	p.Leu587Val						1			
138	c.1937T>C	p.Met646Thr						1			
139	c.2551G>A	p.Val851Met						1			
140	c.2647C>A	p.Pro883Thr						1			

141	c.2647C>A	p.Pro883Thr								1		
142	c.2647C>A	p.Pro883Thr								1		
143	c.2230C>A	p.Pro744Thr										1
143	Duplication of exons 8-14	p.?										1
**Compound heterozygous**												
144	c.86A>C	p.His29Pro						1				
144	c.700C>A	p.Pro234Thr						1				
145	c.180+3A>T	p.?										1
145	c.907T>C	p.Trp303Arg	1									
146	c.180+3A>T	p.?										1
146	c.854G>A	p.Gly285Glu		1								
147	c.180+3A>T	p.?					1					
147	c.568G>A	p.Gly190Arg					1					
148	c.180+3A>T	p.?						1				
148	c.1222C>G	p.Pro408Ala						1				
149	c.180+3A>T	p.?					1					
149	c.501C>G	p.Phe167Leu					1					
150	c.180+3A>T	p.?										1
150	c.854G>A	p.Gly285Glu		1								
151	c.180+3A>T	p.?								1		
151	c.1679T>C	p.Met560Thr								1		
152	c.180+3A>T	p.?								1		
152	c.1748A>G	p.Gln583Arg								1		
153	c.180G>A	p.=						1				
153	c.1918G>T	p.Val640Phe						1				
154	c.209C>T	p.Ser70Leu					1					
154	c.920T>C	p.Phe307Ser					1					
155	c.220C>T	p.Gln74X								1		
155	c.1477G>A	p.Ala493Thr								1		
156	c.434-2_434dupAGC	p.Ala145dup						1				
156	c.854G>A	p.Gly285Glu						1				
157	c.434-2_434dupAGC	p.Ala145dup						1				
157	c.854G>A	p.Gly285Glu						1				
158	c.434-2_434dupAGC	p.Ala145dup										1
158	c.854G>A	p.Gly285Glu										1
159	c.501C>G	p.Phe167Leu										1
159	c.689G>A	p.Gly230Glu	1									
160	c.501C>G	p.Phe167Leu						1				
160	c.1930+6T>G	p.?						1				
161	c.501C>G	p.Phe167Leu						1				
161	c.1013G>A	p.Arg338Gln						1				
162	c.501C>G	p.Phe167Leu						1				
162	c.1478C>A	p.Ala493Glu						1				
163	c.501C>G	p.Phe167Leu						1				
163	c.2551G>A	p.Val851Met						1				
164	c.501C>G	p.Phe167Leu					1					
164	c.1437_1450del	p.Pro480fs					1					
165	c.501C>G	p.Phe167Leu						1				
165	c.1696G>A	p.Ala566Thr						1				

166	c.501C>G	p.Phe167Leu				1	
166	c.1167-10T>C	p.?				1	
167	c.501C>G	p.Phe167Leu				1	
167	c.1586C>T	p.Ala529Val	F			1	
168	c.568_569delinsTC	p.Gly190Ser			1		
168	c.1453A>G	p.Met485Val			1		
169	c.568_569delinsTC	p.Gly190Ser			1		
169	c.920T>C	p.Phe307Ser			1		
170	c.568_569delinsTC	p.Gly190Ser			1		
170	c.1387T>A	p.Phe463Ile			1		
171	c.568G>A	p.Gly190Arg			1		
171	c.1453A>G	p.Met485Val			1		
172	c.568G>A	p.Gly190Arg			1		
172	c.599G>A	p.Gly200Glu			1		
173	c.592C>G	p.Leu198Val					1
173	c.689G>A	p.Gly230Glu					1
174	c.689G>A	p.Gly230Glu	1				
174	c.1453A>G	p.Met485Val					1
175	c.689G>A	p.Gly230Glu				1	
175	c.1297T>C	p.Trp433Arg				1	
176	c.689G>A	p.Gly230Glu				1	
176	c.1606G>A	p.Val536Ile				1	
177	c.701C>T	p.Pro234Leu					1
177	c.1167-16T>G	p.?					1
178	c.774+1G>A	p.?			1		
178	c.1222C>G	p.Pro408Ala			1		
179	c.803C>T	p.Thr268Met				1	
179	c.979G>A	p.Val327Ile				1	
180	c.803C>T	p.Thr268Met	1				
180	c.1264G>A	p.Glu422Lys					1
181	c.817G>A	p.Val273Met				1	
181	c.1013G>A	p.Arg338Gln				1	
182	c.826G>A	p.Gly276Ser			1		
182	c.1437_1450del	p.Pro480fs			1		
183	c.829T>C	p.Cys277Arg			1		
183	Deletion of exons 1 to 3	p.?			1		
184	c.854G>A	p.Gly285Glu	1				
184	c.1222C>G	p.Pro408Ala					1
185	c.854G>A	p.Gly285Glu			1		
185	c.1748A>G	p.Gln583Arg			1		
186	c.854G>A	p.Gly285Glu			1		
186	c.1453A>G	p.Met485Val			1		
187	c.854G>A	p.Gly285Glu			1		
187	c.1453A>G;	p.Met485Val			1		
188	c.854G>A	p.Gly285Glu			1		
188	c.1453A>G	p.Met485Val			1		
189	c.854G>A	p.Gly285Glu			1		
189	c.1447G>A	p.Gly483Ser			1		
190	c.854G>A	p.Gly285Glu			1		
190	c.1582+1G>A	p.?			1		

191	c.854G>A	p.Gly285Glu			1			
191	c.2680C>T	p.Arg894X			1			
192	c.854G>A	p.Gly285Glu						1
192	c.2680C>T	p.Arg894X						1
193	c.854G>A	p.Gly285Glu					1	
193	c.979G>A	p.Val327Ile					1	
194	c.854G>A	p.Gly285Glu					1	
194	c.1238T>G	p.Phe413Cys					1	
195	c.854G>A	p.Gly285Glu					1	
195	c.1437_1450 del	p.Pro480fs					1	
196	c.854G>A	p.Gly285Glu					1	
196	c.2680C>T	p.Arg894X					1	
197	c.854G>A	p.Gly285Glu						1
197	c.1269dupC	p.Ile424fs						1
198	c.866G>T	p.Ser289Ile			1			
198	c.2680C>T	p.Arg894X			1			
199	c.907T>C	p.Trp303Arg					1	
199	c.1222C>G	p.Pro408Ala					1	
200	c.920T>C	p.Phe307Ser				1		
200	c.1167-10T>C	p.?				1		
201	c.920T>C	p.Phe307Ser				1		
201	c.1437_1450 del	p.Pro480fs				1		
202	c.938C>T	p.Ala313Val						1
202	c.1453A>G	p.Met485Val						1
203	c.938C>T	p.Ala313Val					1	
203	c.1222C>G	p.Pro408Ala					1	
204	c.950G>A	p.Arg317Leu			1			
204	c.1437_1450 del	p.Pro480fs			1			
205	c.959C>T	p.Ala320Val				1		
205	c.1437_1450 del	p.Pro480fs				1		
206	c.991G>T	p.Ala331Ser				1		
206	c.1437_1450 del	p.Pro480fs				1		
207	c.1063G>A	p.Gly355Arg			1			
207	c.2680C>T	p.Arg894X			1			
208	c.1106A>C	p.His369Pro					1	
208	c.1872delG	p.Glu624fs				1		
209	c.1129C>T	p.Arg377X					1	
209	c.1222C>G	p.Pro408Ala					1	
210	c.1205C>T	p.Ala402Val				1		
210	c.1561C>A	p.Pro521Thr				1		
211	c.1261C>T	p.Arg421Cys			1			
211	c.2596-11C>G	p.?			1			
212	c.1437_1450 del	p.Pro480fs				1		
212	c.2464G>A	p.Asp822Asn				1		
213	c.1437_1450 del	p.Pro480fs						1
213	c.1991A>C	p.His664Pro						1
214	c.1437_1450 del	p.Pro480fs					1	
214	c.1438C>T	p.Pro480Ser					1	
215	c.1439C>A	p.Pro480His						1
215	c.1453A>G	p.Met485Val						1

216	c.1439C>A	p.Pro480His										1
216	c.2680C>T	p.Arg894X										1
217	c.1453A>G	p.Met485Val						1				
217	c.1872delG	p.Glu624fs						1				
218	c.1453A>G	p.Met485Val								1		
218	2010_2020del	p.Arg670fs								1		
**More than two variants**												
219	c.461A>G	p.Gln154Arg										1
219	c.1480T>C	p.Phe494Leu										1
219	c.1672C>T	p.Pro558Ser					1					
220	c.313C>T	p.Arg105Cys										1
220	c.501C>G	p.Phe167Leu										1
220	c.1872delG	p.Glu624fs						1				
221	c.316C>G	p.Leu106Val										1
221	c.1649C>T	p.Thr550Met										1
221	c.2596-1G>A	p.?										1
222	c.501C>G	P.Phe167Leu										1
222	c.568G>A	p.Gly190Arg										1
222	c.1453A>G	p.Met485Val										1
223	c.664G>A	p.Gly222Ser										1
223	c.1672C>T	p.Pro558Ser					1					
223	c.2926C>T	p.Arg976X										1
			27	6	48	21	25	74	18	45	6	39

Supplementary Table 1. Inheritance patterns of ClC-1 variants in our cohort for each pedigree. Sp: sporadic, SC: segregation confirmed, VP: variable penetrance, SR: segregation reported. When the inheritance was unknown or the association with MC was uncertain it is specified if the variants were found in heterozygosis (Het) or compound heterozygosis (CH). Homozygous variants are included in the CH column.

	Dominant			Sp	Recessive		Unknown		Uncertain		
	SC	VP	SR		SC	SR	Het	CH	Het	CH	Sum
**p.His29Pro**						1					1
**p.Ser70Leu**					1						1
**p.Arg105Cys**										1	1
**p.Leu106Val***										1	1
**p.Met128Ile**	1		1								2
**p.Gln154Arg**										1	1
**p.Phe167Leu**					2	5		2	1	3	13
**p.Cys179Tyr***			1								1
**p.Ser183Pro**			1								1
**p.Gly190Arg**					1	2				1	4
**p.Gly190Ser**						3					3
**p.Leu198Pro**							1				1
**p.Leu198Val**										1	1
**p.Gly200Glu***						1					1
**p.Ala218Val**			1								1
**p.Ala221Glu***						1					1
**p.Gly222Ser**										1	1
**p.Gly230Glu**	11	1	17	5			3	2		1	40
**p.Gly233Ser**					1						1
**p.Pro234Thr***						1					1
**p.Pro234Leu***										1	1
**p.Thr268Met**		1					1	1			3
**p.Cys271Arg**	1										1
**p.Val273Met**								1			1
**p.Gly276Asp**	2										2
**p.Gly276Ser**						1					1
**p.Cys277Arg**						1					1
**p.Gly285Glu**	1	2	2	3	1	8	1	4	1	3	26
**p.Gly285Val***	1										1
**p.Ser289Gly***		1									1
**p.Ser289Asn**			1	1							2
**p.Ser289Ile***					1						1
**p.Glu291Lys**					1						1
**p.Phe297Ser**	2		5								7
**p.Val299Leu**					1	1					2
**p.Trp303Arg**	3		9	2			1	1			16
**p.Phe306Leu**		1	1	1			2				5
**p.Phe307Ser**			1	2	1	3	2	1			10
**p.Ala313Thr**	3		6	1		1	1				12
**p.Ala313Val**	1		1					1	1	1	5
**p.Arg317Leu**					1						1
**p.Ala320Val**						1					1
**p.Val321Leu**				1							1
**p.Val327Ile**								2			2
**p.Thr328Ile**			1				1	3			5
**p.Ala331Ser***						1					1
**p.Leu332Arg***				1							1
**p.Phe333Leu***					1						1
**p.Arg338Gln**						1		1			2
**p.Pro342Leu***				1							1

**p.Gly355Arg**					1						1
**p.His369Pro**								1			1
**p.Val397Asp***								1			1
**p.Ala402Val**				1		1					2
**p.Pro408Ala**						2		3		1	6
**p.Phe413Cys**								1			1
**p.Arg421Cys**					1						1
**p.Glu422Lys**										1	1
**p.Trp433Arg**								1			1
**p.Val456Ile***							1				1
**p.Phe463Ile***						1					1
**p.Pro480His**										2	2
**p.Pro480Ser**	1							1			2
**p.Gly483Ser***						1					1
**p.Met485Val**						6		1		4	11
**p.Met485Lys***						1					1
**p.Ala493Glu**						1					1
**p.Ala493Thr***								1			1
**p.Phe494Leu***										1	1
**p.Glu500Lys***						1					1
**p.Pro521Thr***						1					1
**p.Gly523Asp**				1							1
**p.Ala529Val***								1			1
**p.Val536Ile**								1			1
**p.Glu548Lys**								1			1
**p.Thr550Met**										1	1
**p.Gly551Asp**							1				1
**p.Pro558Ser**					2						2
**p.Met560Thr**								1			1
**p.Ala566Thr**					1	1					2
**p.Gln583Arg***						1		1			2
**p.Leu587Val***						2					2
**p.Gly594Val***							1				1
**p.Arg611His***							1		1		2
**p.Val640Phe***						1					1
**p.Met646Thr***						1					1
**p.His664Pro***										1	1
**p.Arg669Cys**				1							1
**p.Gly688Arg***							1				1
**p.Pro744Thr**										1	1
**p.Asp822Asn***						1					1
**p.Thr837Ile***									1		1
**p.Val851Met**						2					2
**p.Pro883Thr**						1		2			3
**p.Gly898Arg***									1		1
**Total**	27	6	48	21	17	57	18	36	6	27	263

Supplementary table 2. Inheritance patterns of ClC-1 missense variants. Dom: Dominant, Spor: sporadic, Rec: recessive inheritance. SC: segregation confirmed, VP:

variable penetrance, SR: segregation reported. When the inheritance was unknown or the association with MC was uncertain it is specified if the variants were found in heterozygosis (Het) or compound heterozygosis (CH). Asterisks indicate variants not reported in the literature previously. Data for the variants that were not missense are presented in Supplementary table 3.

Nucleotide	Protein	Dominant			Sp	Recessive		Unknown		Uncertain		Total
		SC	VP	SR		SC	SR	Het	CH	Het	CH	
**c.180G>A***	p.=						1					1
**c.180+3A>T**	p.?					2	1		2		3	8
**c.220C>T**	p.Gln74X								1			1
**c.434-2_434dupAGC**	p.Ala145dup						2				1	3
**c.774+1G>A**	p.?						1					1
**c.1129C>T**	p.Arg377X								1			1
**c.1167-10T>C**	p.?						1		1			2
**c.1167-16T>G***	p.?										1	1
**c.1269dupC***	p.Ile424fs										1	1
**c.1437_1450 del**	p.Pro480fs					2	5		2		1	10
**c.1582+1G>A***	p.?						1					1
**c.1872delG**	p.Glu624fs						3					3
**c.1930+6T>G***	p.?						1					1
**c.2010_2020del***	p.Arg670fs								1			1
**c.2596-1G>A**	p.?										1	1
**c.2596-11C>G**	p.?					1						1
**c.2680C>T**	p.Arg894X					3			1		2	6
**c.2926C>T**	p.Arg976X										1	1
**Duplication exons 8-14**	Duplication exons 8-14										1	1
**Deletion exons 1-3***	Deletion exons 1-3						1					1
**Total**						8	17		9		12	46

Supplementary Table 3. Inheritance patterns of ClC-1 variants other than missense in our cohort. Sp: sporadic, SC: segregation confirmed, VP: variable penetrance, SR: segregation reported. When the inheritance was unknown or the association with MC was uncertain it is specified if the variants were found in heterozygosis (Het) or compound heterozygosis (CH). Asterisks indicate variants not reported in the literature to our knowledge previously.

Variant	Homomeric					Simulated heterozygous											Category
	N	$V_{1/2}$ (mV)	SEM	V_{slope} (mV)	SEM	n	$V_{1/2}$ (mV)	SEM	V_{slope} (mV)	SEM	$V_{1/2}$ (mV)	SEM	V_{slope} (mV)	SEM	Frac	SEM	
His29Pro	14	-42.9	2.5	27.2	0.9												WT-like
Ser70Leu	6	-31.7	3.1	24.4	0.9												WT-like
Arg105Cys	5	-28.2	5.2	27.7	1.6												WT-like
Leu106Val	7	-25.4	3.4	25.2	1.2												WT-like
Met128Ile	6	54.7	4.7	33.4	1.4	6	7.0	2.7	33.8	1.4							Dominant
Gln154Arg	7	-29.5	3.4	24.5	1.2												WT-like
Phe167Leu	9	-17.7	4.0	30.4	1.0	4	-26.4	2.5	29.8	3.1							Recessive
Cys179Tyr	6	114.0	6.6	32.1	3.2	7	4.7	5.7	34.4	1.6							Dominant
Ser183Pro	8	-				11	83.2	7.6	39.6	2.1	-35.6	5.9	26.2	2.4	0.33	0.04	Dominant
Gly190Arg	8	-				7	-38.4	1.7	29.1	0.6							Recessive
Gly190Ser	8	118.2	6.6	38.8	1.0	8	53.1	10.4	41.3	1.1							Dominant
Leu198Pro	8	80.2	4.2	36.8	0.8	10	34.9	6.7	35.0	0.8							Dominant
Leu198Val	15	-10.3	2.2	27.8	1.0	12	-23.5	3.4	28.2	0.9							Recessive
Gly200Glu	6	8.1	2.4	26.7	0.8	5	-9.6	6.9	30.4	1.8							Dominant
Ala218Val	11	83.9	6.7	37.5	0.7	9	27.6	10.9	38.3	1.1							Dominant
Ala221Glu	11(5)	-35.6	5.3	26.5	1.6	8	-36.2	4.5	28.6	0.6							Recessive
Gly222Ser	17	-21.1	2.0	27.4	1.0												WT-like
Gly230Glu	9	-				16	74.4	3.0	46.5	1.3	-33.9	1.5	24.7	0.7	0.47	0.03	Dominant
Gly233Ser	7	-				11	-35.6	2.3	24.0	0.7							Recessive
Pro234Leu	11	-				5	55.8	10.9	48.5	3.7	-35.6	3.8	24.2	1.9	0.50	0.05	Dominant
Pro234Thr	8	65.4	2.8	36.7	1.2	9	-1.6	2.1	32.9	0.7							Dominant
Thr268Met	6	48.0	6.5	30.8	0.9	7	-3.3	4.3	29.5	0.8							Dominant
Cys271Arg	5	125.4	8.1	44.8	1.9	6	35.8	4.2	43.4	1.5							Dominant
Val273Met	12	4.9	3.0	27.3	0.7	9	-25.7	4.1	25.8	1.0							Recessive
Gly276Asp	9	-				10	75.0	7.7	46.7	2.8	-25.5	2.7	20.4	1.1	0.38	0.05	Dominant
Gly276Ser	14	-12.3	1.4	32.8	0.9	11	-19.2	2.5	28.8	1.5							Recessive
Cys277Arg	10	-				7	-27.0	3.2	27.2	1.1							Recessive
Gly285Glu	12	-				13	117.5	3.5	45.9	2.7	-30.4	2.7	20.5	0.9	0.40	0.05	Dominant
Gly285Val	9	-				7	-26.3	4.5	26.3	1.7							Recessive
Ser289Asn	5	104.6	3.1	34.8	1.1	8	18.6	5.6	38.0	1.1							Dominant
Ser289Gly	6	61.6	5.8	32.9	0.4	5	13.1	3.5	34.0	0.8							Dominant
Ser289Ile	6	124.1	4.4	37.4	2.1	11	9.4	8.0	39.7	3.7							Dominant
Glu291Lys	8	-				7	-27.1	5.5	25.2	1.4							Recessive
Phe297Ser	14	-6.1	2.5	27.6	0.9	14	-0.4	3.6	32.8	0.8							Dominant
Val299Leu	9	3.3	2.9	26.8	1.0	12	-9.2	3.7	27.4	1.0							Dominant
Trp303Arg	7(4)	75.5	3.4	38.8	2.4	11	69.4	2.3	46.1	3.8	-42.2	5.3	19.9	1.3	0.34	0.07	Dominant
Phe306Leu	7	88.9	3.4	37.7	1.3	6	13.2	3.5	38.3	1.0							Dominant
Phe307Ser	6	15.2	4.2	26.4	2.2	6	-8.4	2.3	28.8	1.0							Dominant
Ala313Thr	16	61.2	4.1	33.1	1.2	21	-11.5	2.8	32.5	1.0							Dominant
Ala313Val	5	72.6	2.6	31.2	1.2	6	24.3	4.0	35.8	1.3							Dominant
Arg317Leu	7	5.8	2.6	32.1	0.7	8	-32.8	3.0	34.6	3.3							Recessive
Ala320Val	8	-4.9	2.6	36.7	2.0	7	-31.8	3.5	30.4	0.9							Recessive
Val321Leu	6	95.5	12.9	51.3	2.1	13	-3.3	3.4	37.3	1.1							Dominant
Val327Ile	8	-43.5	7.3	25.1	1.6												WT-like
Thr328Ile	10	-				8	85.5	4.9	47.2	3.4	-33.8	4.8	22.1	1.5	0.41	0.02	Dominant
Ala331Ser	27	-19.6	1.6	32.8	0.7												WT-like
Leu332Arg	13	*				8	-18.9	2.8	42.7	1.4							Other
Phe333Leu	13	-25.5	3.0	32.7	1.0												WT-like
Arg338Gln	26	-8.5	3.6	33.4	0.9	10	-24.5	2.1	29.7	1.8							Recessive
Pro342Leu	11	*				6	-20.5	4.0	36.3	0.9							Other
Gly355Arg	8	-				8	-31.5	2.9	25.0	0.7							Recessive
His369Pro	14(10)	-40.4	4.0	26.9	1.8	6	-27.1	2.4	24.3	0.8							Recessive
Val397Asp	31(11)	-23.0	2.5	24.3	1.1	10	-24.8	1.9	27.6	1.8							Recessive
Ala402Val	11	-24.2	2.7	28.1	0.8												WT-like
Pro408Ala	10	-40.9	3.5	32.6	2.2												WT-like

Phe413Cys	19(9)	-29.3	3.5	22.6	1.4	6	-35.8	3.2	22.1	0.9							Recessive
Arg421Cys	11	18.7*	4.0	38.4	1.5	15	-25.5	2.6	29.3	1.2							Other
Glu422Lys	28(19)	-31.4	2.0	24.1	0.7	6	-34.3	6.0	28.8	2.2							Recessive
Trp433Arg	9	-				6	-38.0	2.6	27.9	1.0							Recessive
Val456Ile	9	-38.0	1.9	25.8	1.0												WT-like
Phe463Ile	15	-				11	-31.6	2.2	26.0	1.0							Recessive
Pro480His	6	-				8	68.6	5.4	35.7	2.1	-38.1	4.0	24.8	3.6	0.40	0.07	Dominant
Pro480Ser	12	2.3	3.9	37.3	0.7	13	-13.7	2.3	30.9	0.8							Dominant
Gly483Ser	15	-0.5	2.6	31.5	0.7	9	-23.0	2.8	25.5	0.7							Recessive
Met485Lys	14	86.0*	9.7	53.0	4.5	12	-4.7	3.3	41.1	1.5							Other
Met485Val	12	-				6	-32.6	1.9	26.6	1.9							Recessive
Ala493Glu	16	-				10	-23.8	3.8	25.8	1.1							Recessive
Ala493Thr	10	-32.1	4.5	27.9	1.1												WT-like
Phe494Leu	12	-24.8	2.3	24.5	0.7												WT-like
Glu500Lys	11	-				11	-24.1	2.7	25.0	0.8							Recessive
Pro521Thr	13	-				12	-27.5	2.7	25.4	1.4							Recessive
Gly523Asp	6	110.5	3.7	43.4	1.3	7	7.0	7.0	40.1	2.2							Dominant
Ala529Val	13	-				13	-26.1	3.0	26.7	1.1							Recessive
Val536Ile	6	13.1	6.5	33.0	1.4	6	-15.0	4.3	31.4	1.0							Dominant
Glu548Lys	6	-				7	-32.1	3.3	24.4	1.2							Recessive
Thr550Met	13	-				7	-31.3	2.1	26.1	1.0							Recessive
Gly551Asp	6	126.3	3.8	34.7	0.5	9	13.5	4.4	37.7	0.7							Dominant
Pro558Ser	8	-				8	-24.0	2.5	24.3	0.7							Recessive
Met560Thr	8	-8.4	5.2	37.0	0.8	9	-24.3	4.5	32.5	1.4							Recessive
Ala566Thr	9	-				12	-28.8	2.1	23.6	1.0							Recessive
Gln583Arg	11	-8.0	2.3	29.2	0.9	6	-36.1	4.0	29.1	2.2							Recessive
Leu587Val	24	-30.7	1.9	22.7	0.5												WT-like
Gly594Val	10	-25.1	1.1	24.2	0.8												WT-like
Arg611His	10	-30.1	2.7	26.4	1.3												WT-like
Val640Phe	13	-				11	-31.5	3.5	26.2	1.4							Recessive
Met646Thr	24	-30.0	1.7	24.5	0.9												WT-like
His664Pro	15	-50.7	2.4	25.7	1.0												WT-like
Arg669Cys	6	-37.8	3.7	24.6	1.0												WT-like
Gly688Arg	6	-28.7	4.1	21.8	1.0												WT-like
Pro744Thr	11	-32.5	3.0	26.0	0.7												WT-like
Asp822Asn	13	-10.3	3.8	33.3	1.4	10	-20.6	3.2	31.0	1.6							Recessive
Thr837Ile	6	-32.9	4.0	26.7	1.1												WT-like
Val851Met	10	-33.2	2.3	26.7	1.1												WT-like
Pro883Thr	13	-4.5	2.1	26.3	1.3	14	-21.3	2.5	31.4	1.4							Recessive
Gly898Arg	14	-47.9	3.0	25.1	1.2												WT-like
WT	308	-34.2	0.6	26.0	0.2												

Supplementary table 4. Voltage dependence of activation of ClC-1 variants. First and second column show $V_{1/2}$ and V_{Slope} of the variants in homomeric and simulated heterozygous condition, respectively. Heterozygous condition was simulated by injecting a 50:50 mixture of mRNA encoding WT and mutant subunits. If double Boltzmann equation was used to fit the voltage dependence data, the $V_{1/2}$ and V_{Slope} of the WT-like component is shown in third column. Frac shows the fraction of WT-like component of total amplitude. For some variants two numbers are given in n column.

First number indicates the number of cells tested and number in parenthesis shows the number of cells where current amplitude was sufficient to be fitted with Boltzmann equation. No value is given for a variant in homomeric condition when the current amplitude was too low to allow assessment of $V_{1/2}$. No value is given for a variant in simulated heterozygous condition when the properties of the variant channel in homomeric condition were WT-like. Asterisks denote extraordinary variants (see figure 2).
